# Supplementary figures and images for: DLX1 acts as a novel prognostic biomarker involved in immune cell infiltration and tumor progression in lung adenocarcinoma
Source: PeerJ. 2024 Feb 2;12:e16823. doi: 10.7717/peerj.16823 (PMC10840498; doi:10.7717/peerj.16823)

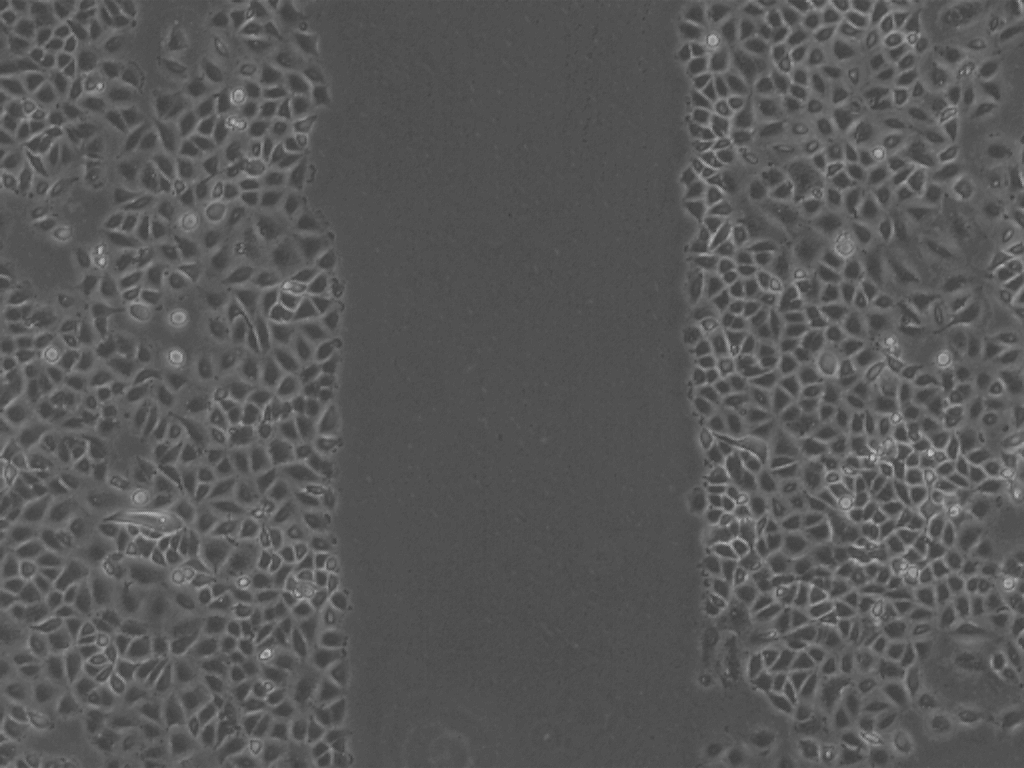

Supplement: Supplemental Information 4 [file peerj-12-16823-s004.zip › A549/0h/D2-1.bmp]

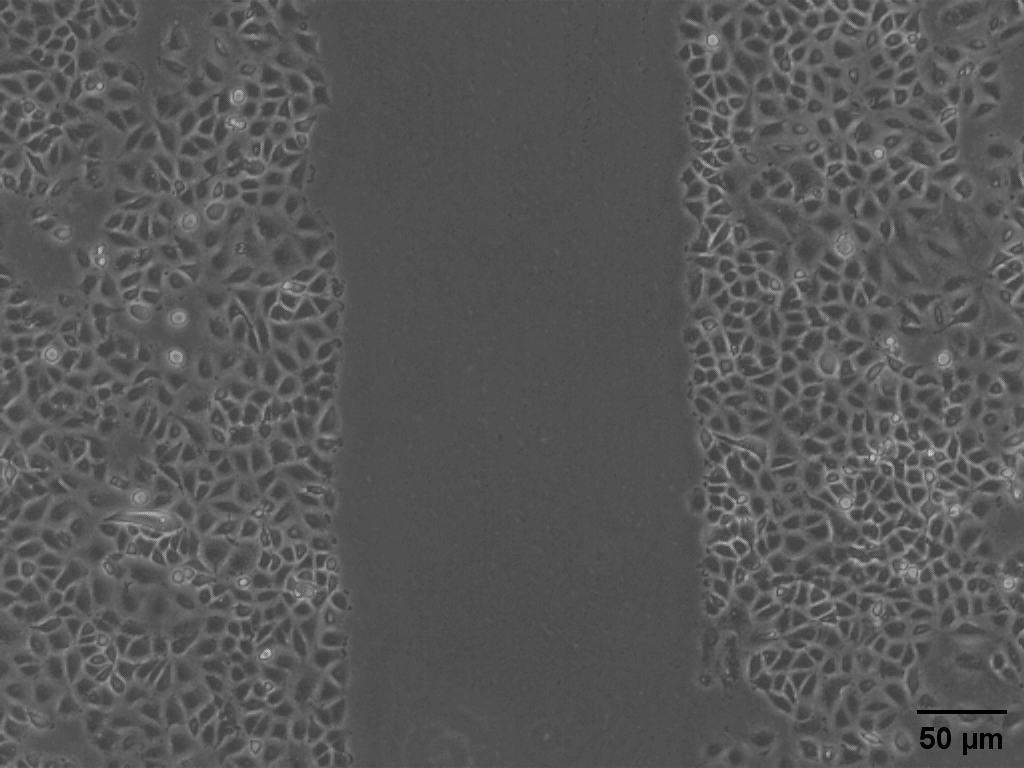

Supplement: Supplemental Information 4 [file peerj-12-16823-s004.zip › A549/0h/D2-1标尺.tif]

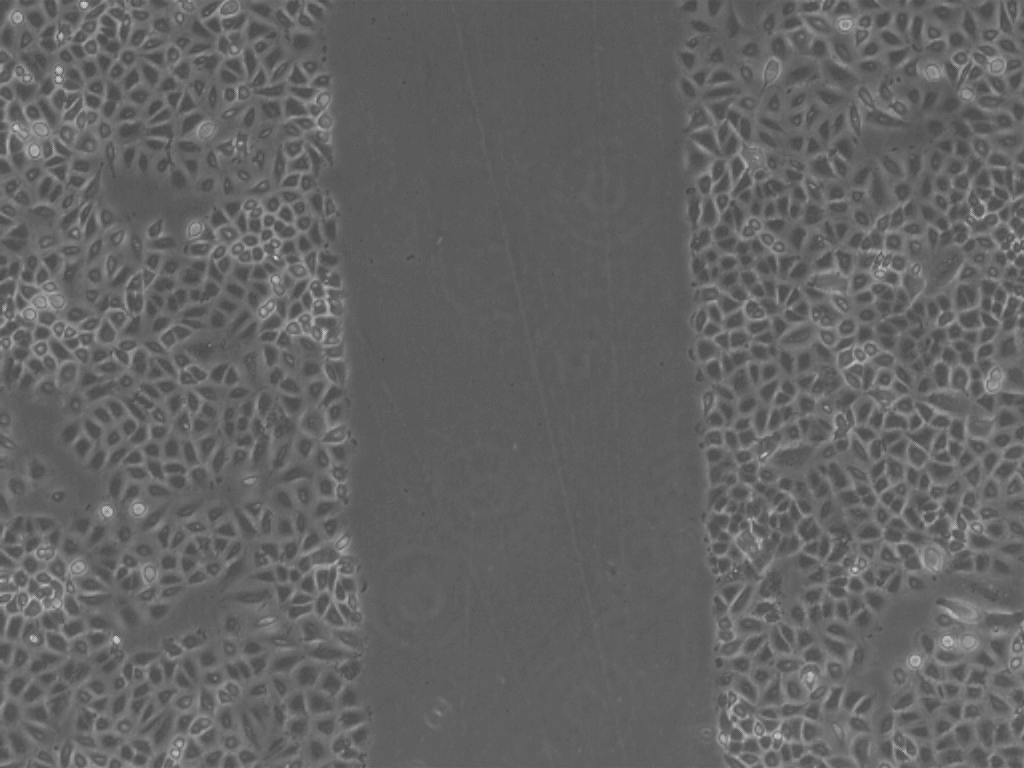

Supplement: Supplemental Information 4 [file peerj-12-16823-s004.zip › A549/0h/D2-2.bmp]

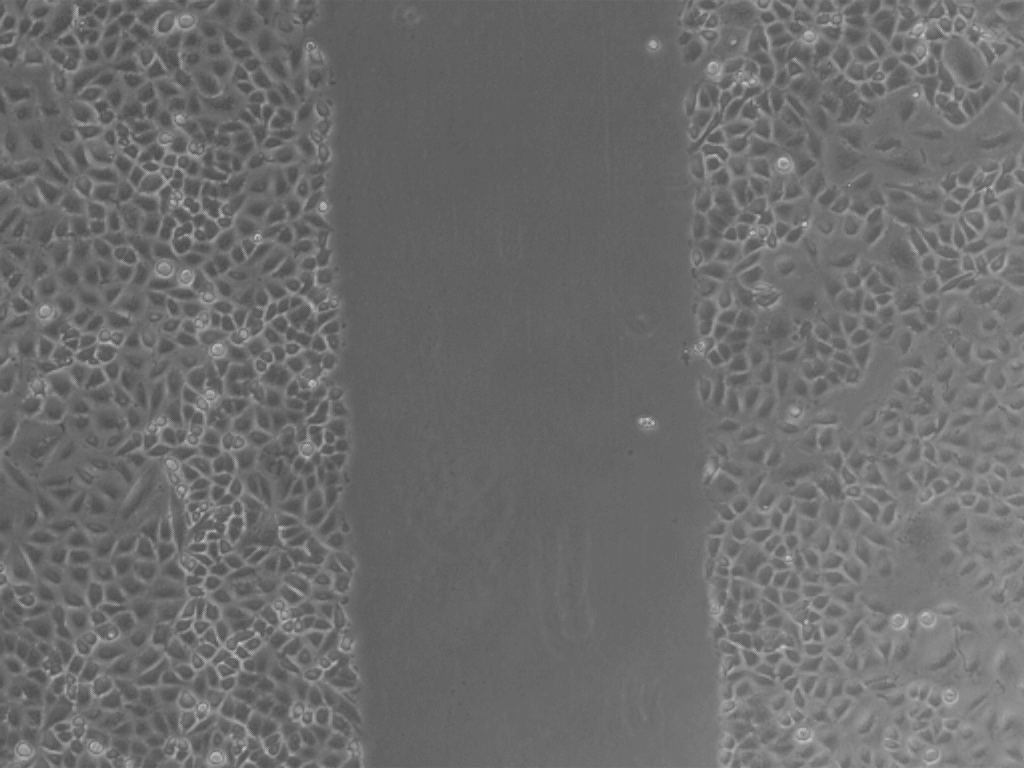

Supplement: Supplemental Information 4 [file peerj-12-16823-s004.zip › A549/0h/D2-3.bmp]

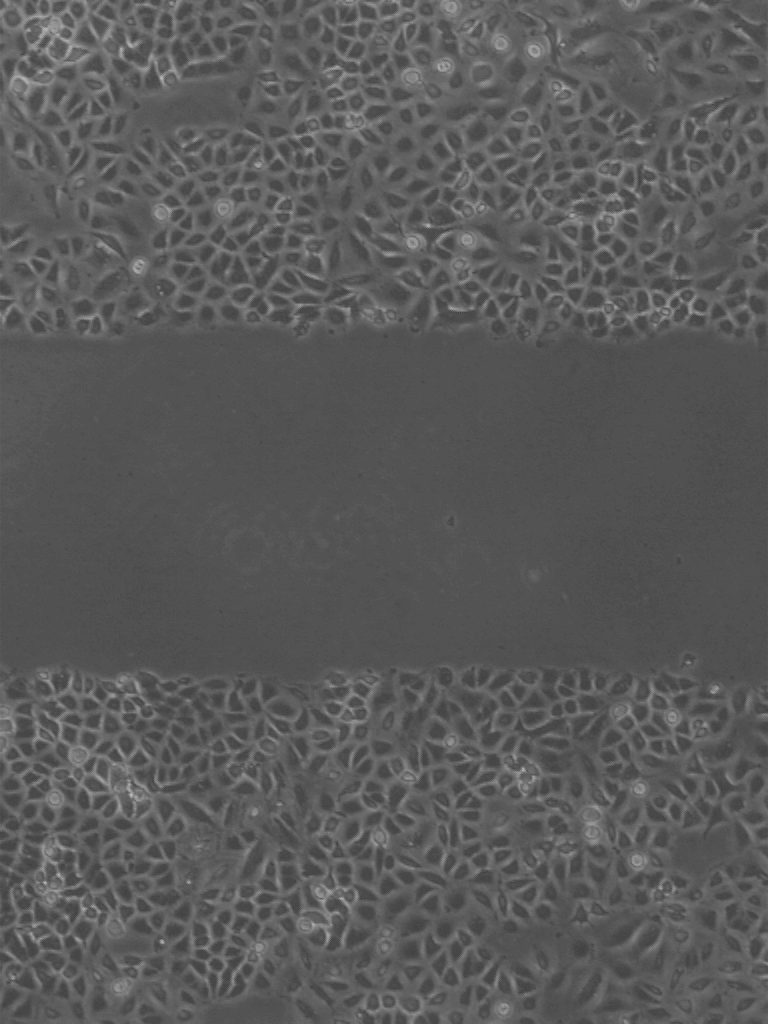

Supplement: Supplemental Information 4 [file peerj-12-16823-s004.zip › A549/0h/D6-1.bmp]

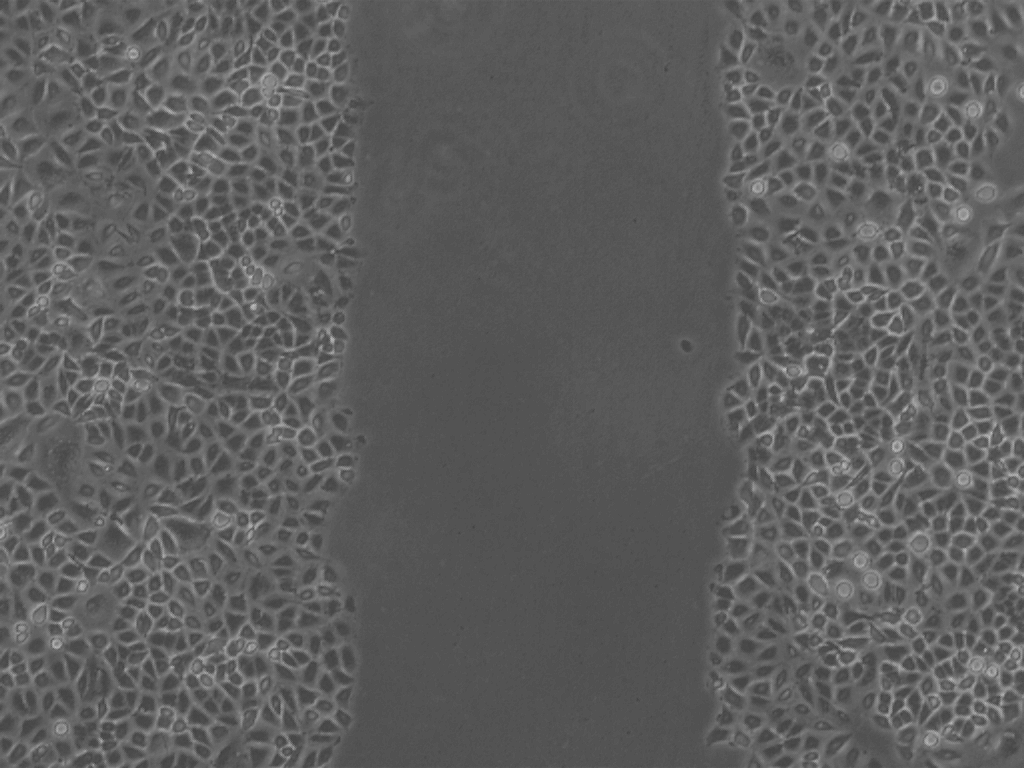

Supplement: Supplemental Information 4 [file peerj-12-16823-s004.zip › A549/0h/D6-2.bmp]

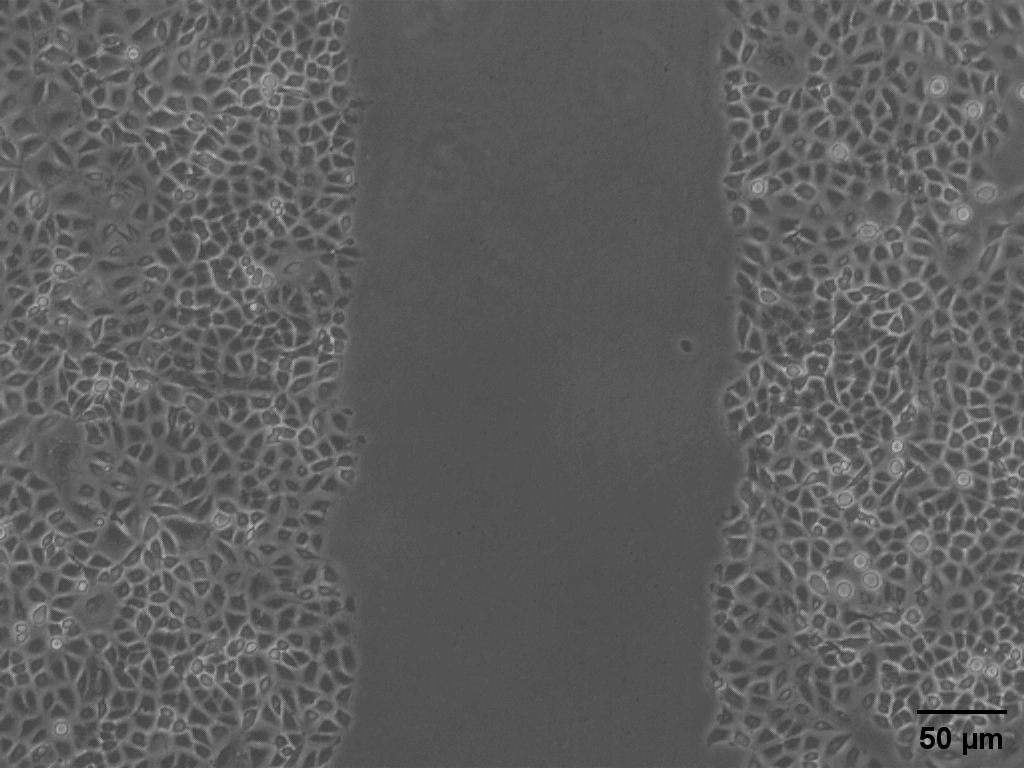

Supplement: Supplemental Information 4 [file peerj-12-16823-s004.zip › A549/0h/D6-2标尺.tif]

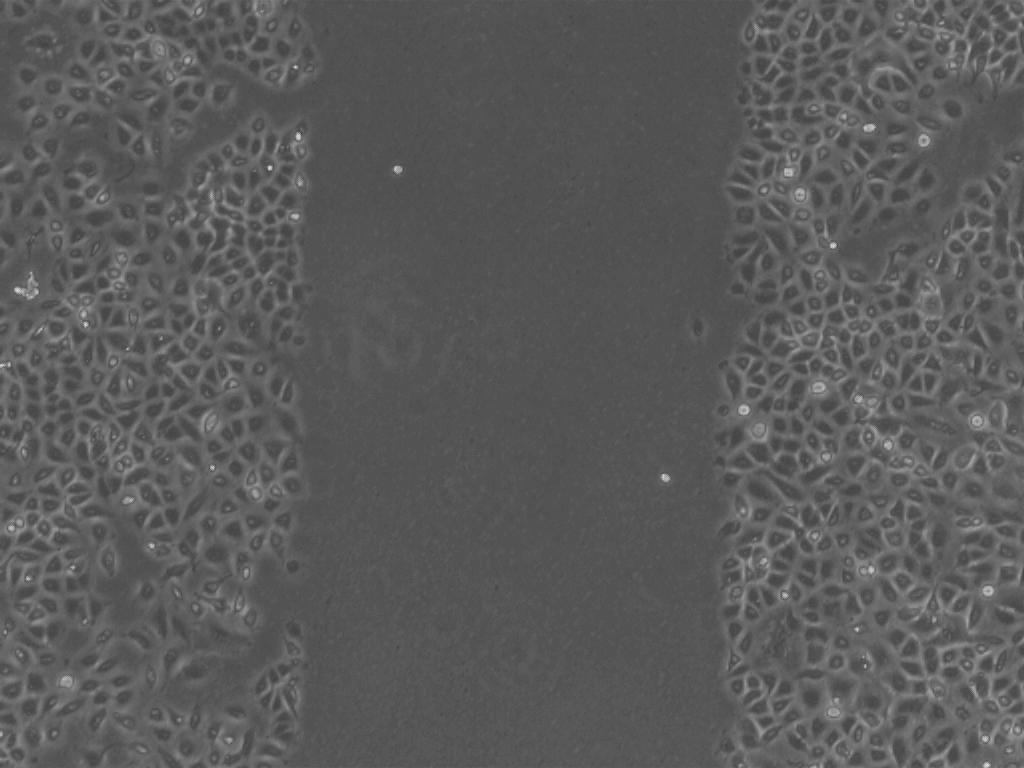

Supplement: Supplemental Information 4 [file peerj-12-16823-s004.zip › A549/0h/D6-3.bmp]

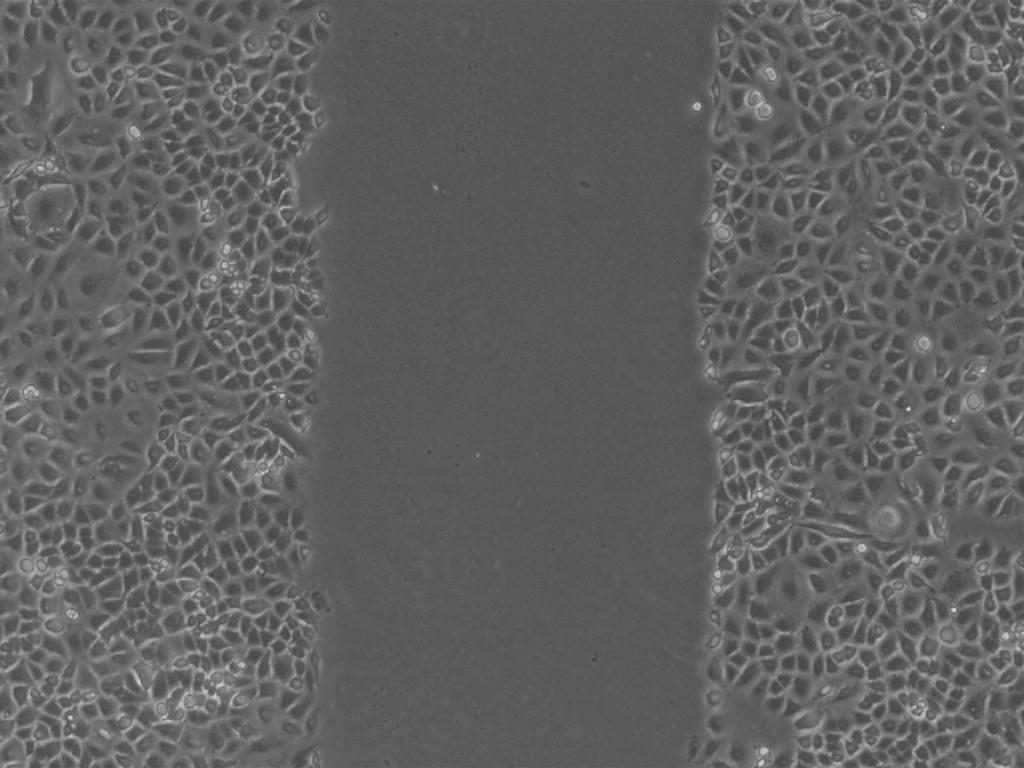

Supplement: Supplemental Information 4 [file peerj-12-16823-s004.zip › A549/0h/NC-1.bmp]

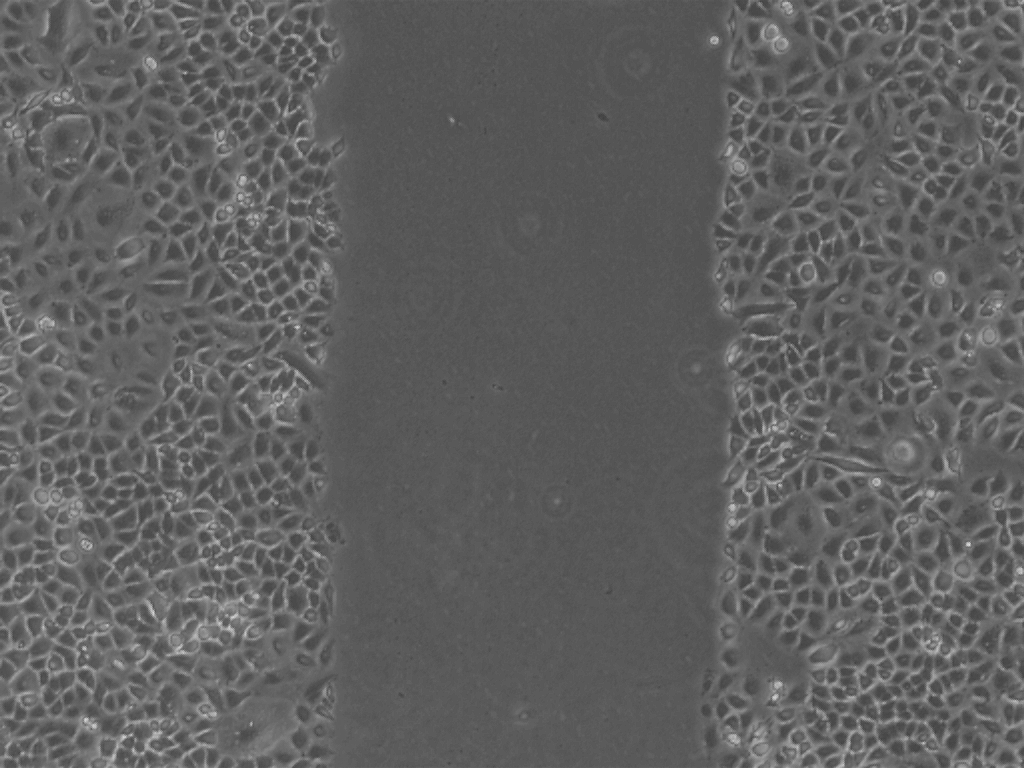

Supplement: Supplemental Information 4 [file peerj-12-16823-s004.zip › A549/0h/NC-2.bmp]

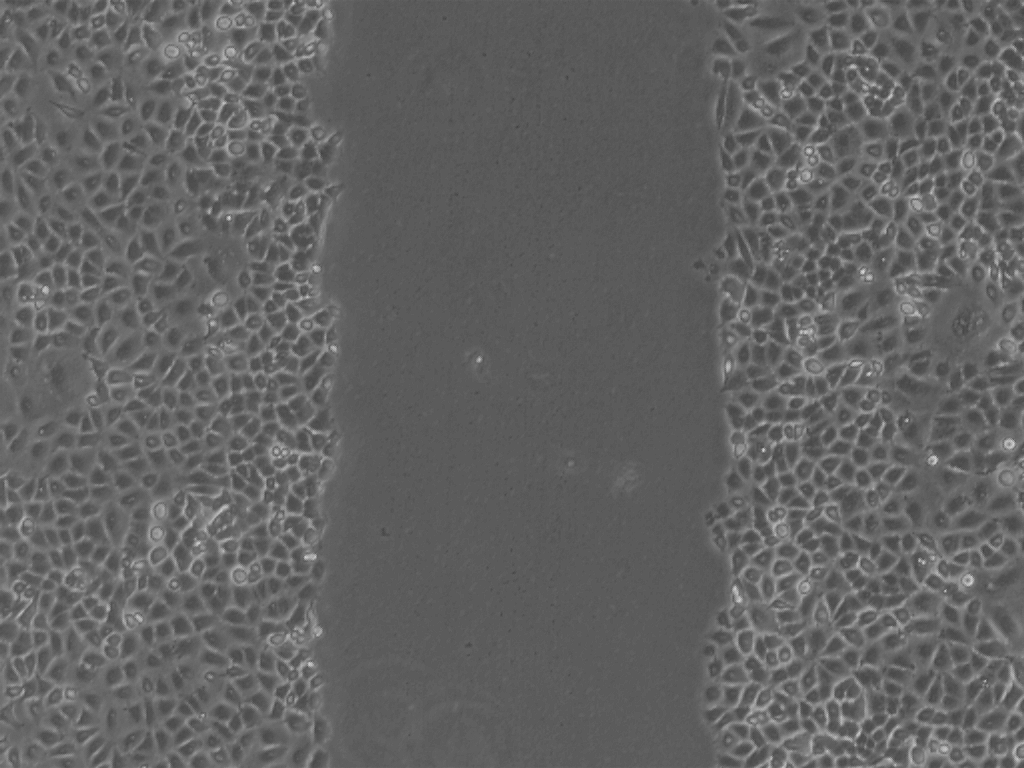

Supplement: Supplemental Information 4 [file peerj-12-16823-s004.zip › A549/0h/NC-3.bmp]

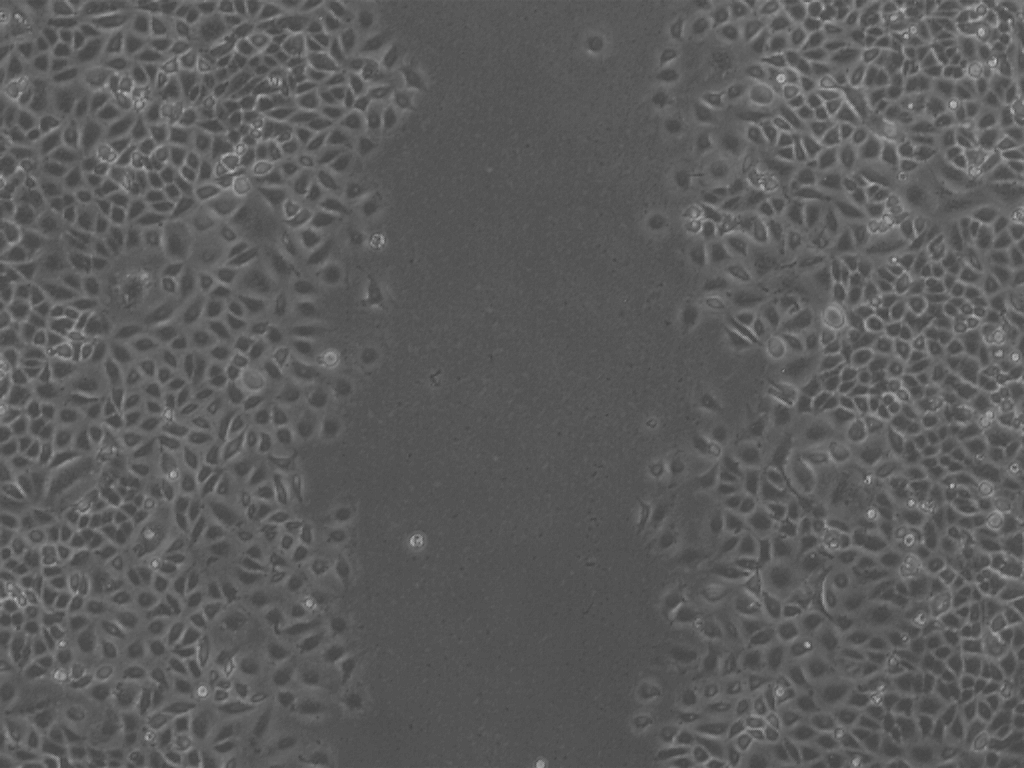

Supplement: Supplemental Information 4 [file peerj-12-16823-s004.zip › A549/24h/D2-1.bmp]

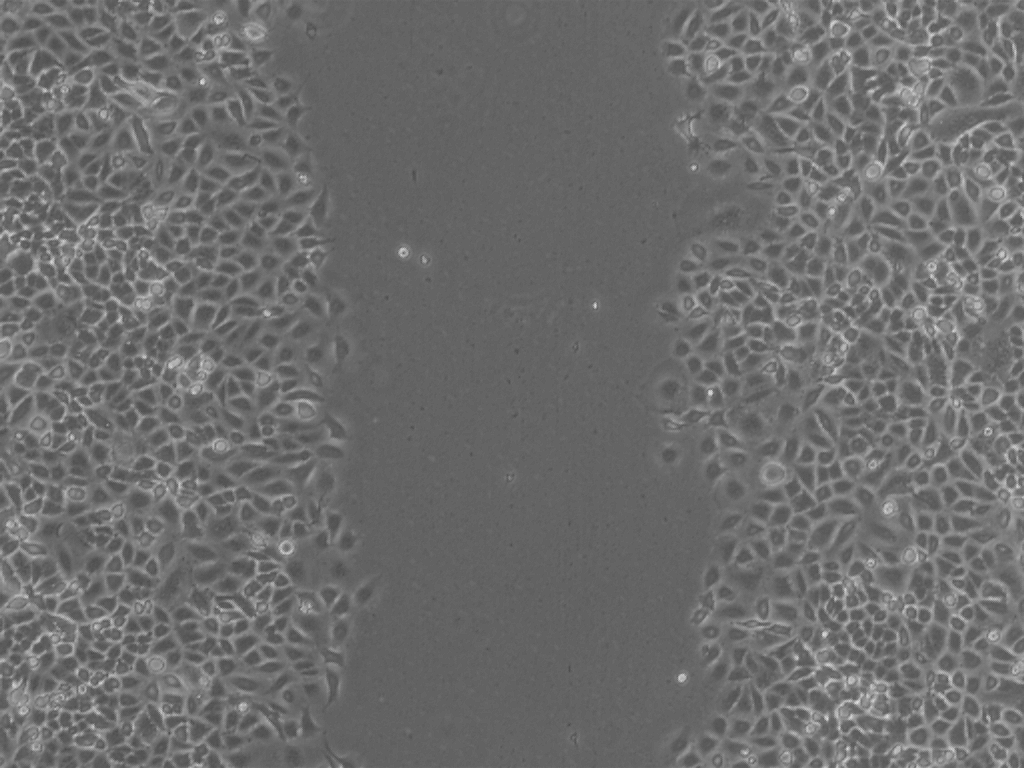

Supplement: Supplemental Information 4 [file peerj-12-16823-s004.zip › A549/24h/D2-2.bmp]

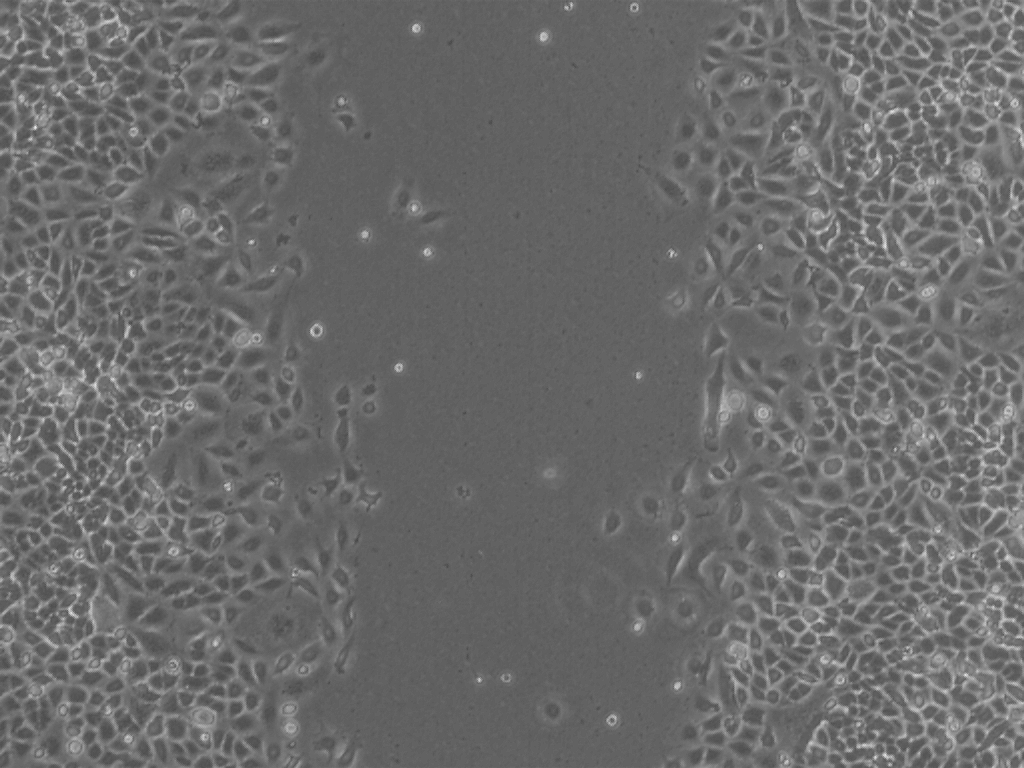

Supplement: Supplemental Information 4 [file peerj-12-16823-s004.zip › A549/24h/D2-3.bmp]

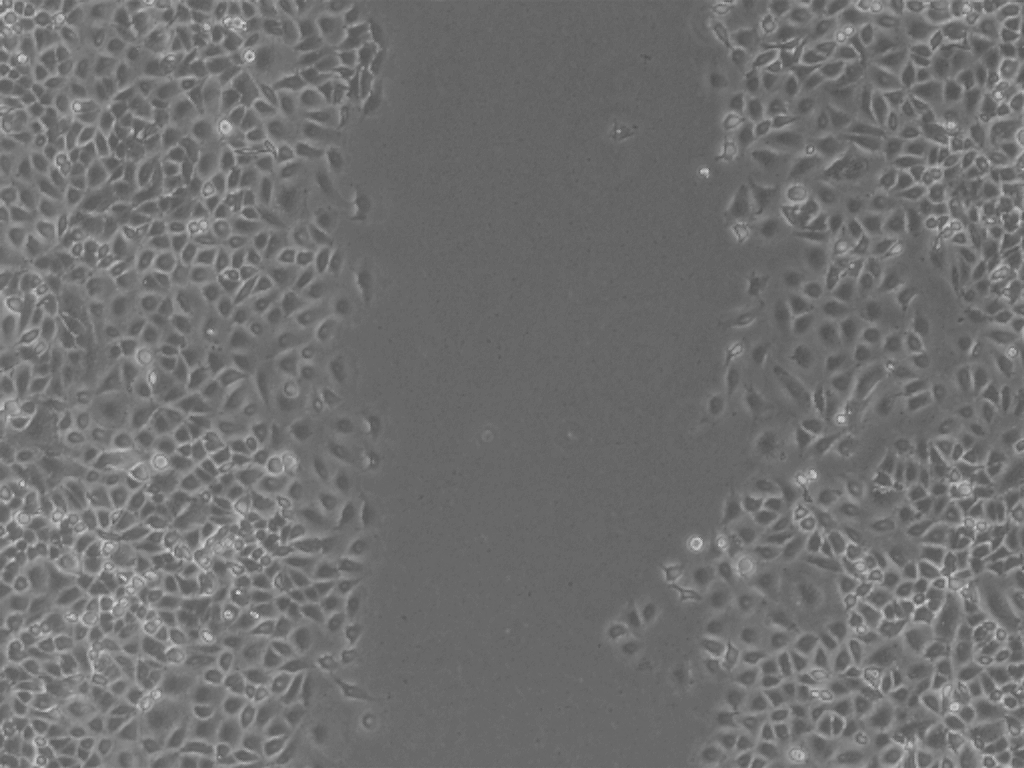

Supplement: Supplemental Information 4 [file peerj-12-16823-s004.zip › A549/24h/D6-1.bmp]

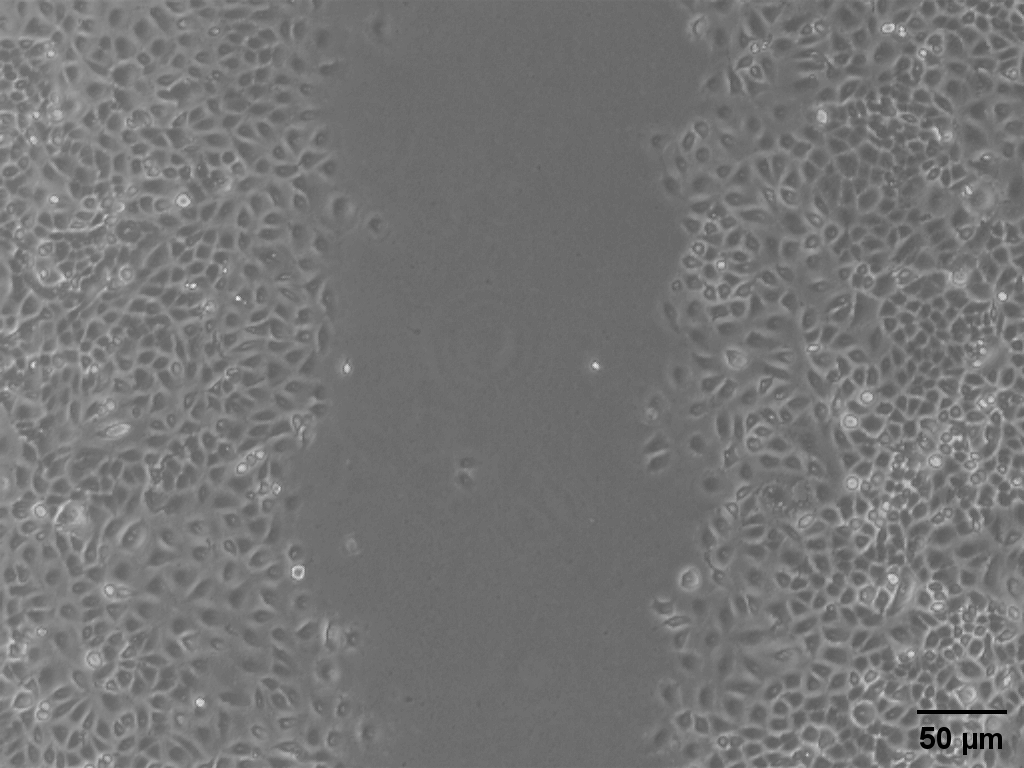

Supplement: Supplemental Information 4 [file peerj-12-16823-s004.zip › A549/24h/D6-2标尺.tif]

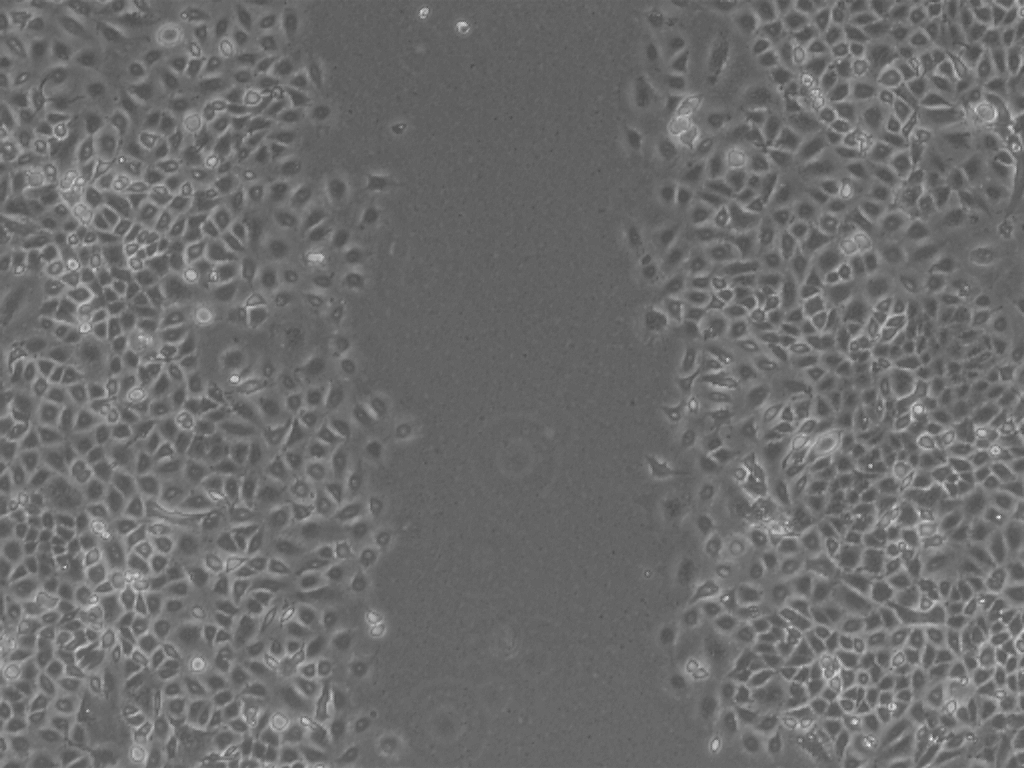

Supplement: Supplemental Information 4 [file peerj-12-16823-s004.zip › A549/24h/D6-3.bmp]

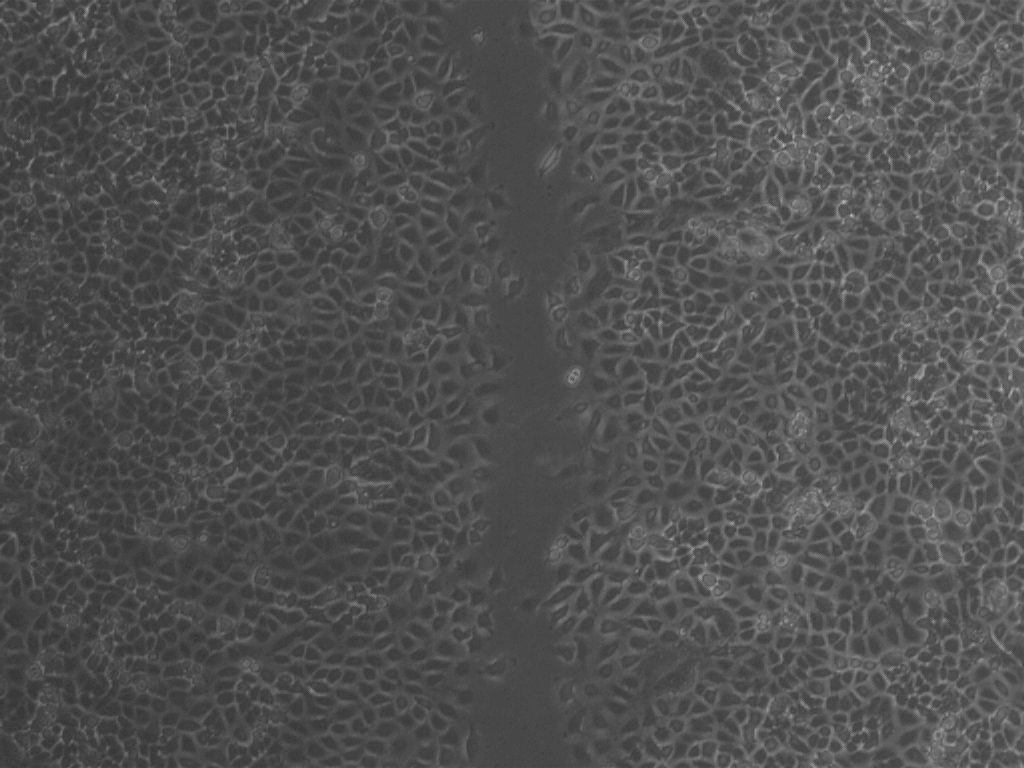

Supplement: Supplemental Information 4 [file peerj-12-16823-s004.zip › A549/24h/NC-1.bmp]

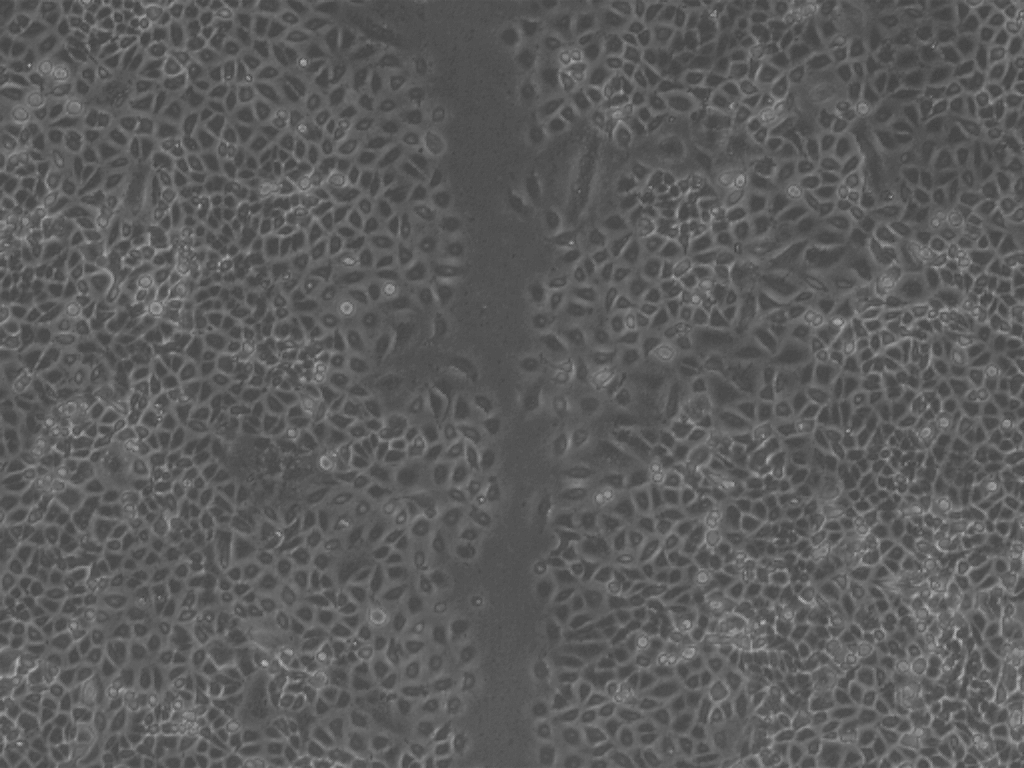

Supplement: Supplemental Information 4 [file peerj-12-16823-s004.zip › A549/24h/NC-2.bmp]

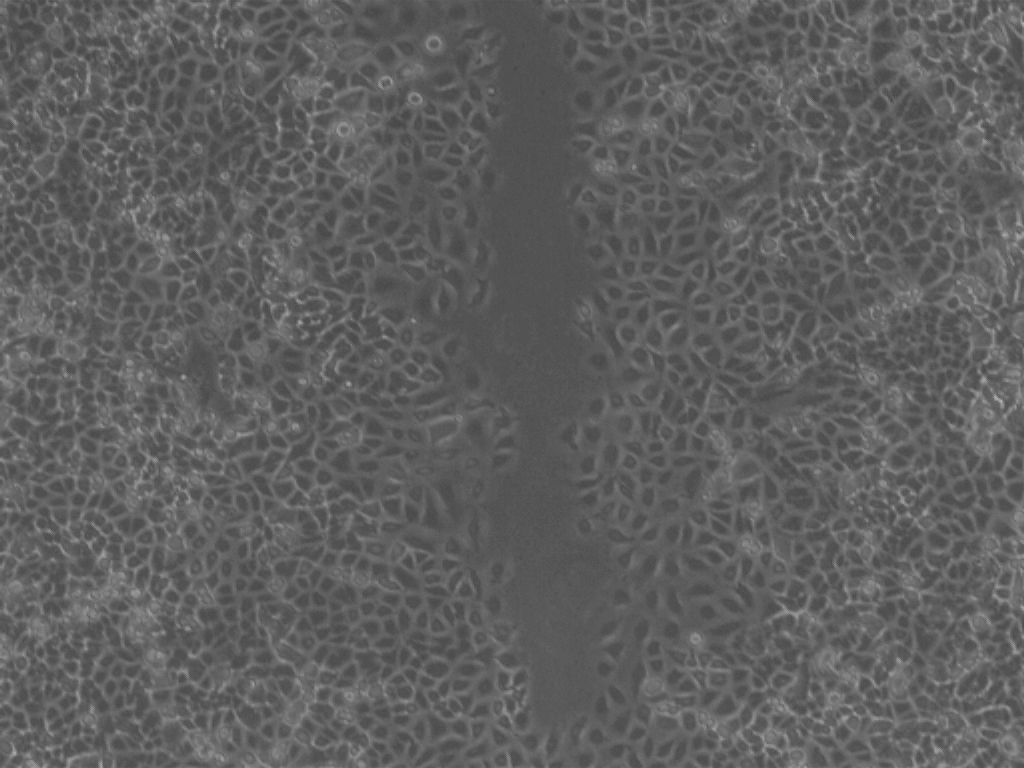

Supplement: Supplemental Information 4 [file peerj-12-16823-s004.zip › A549/24h/NC-3.bmp]

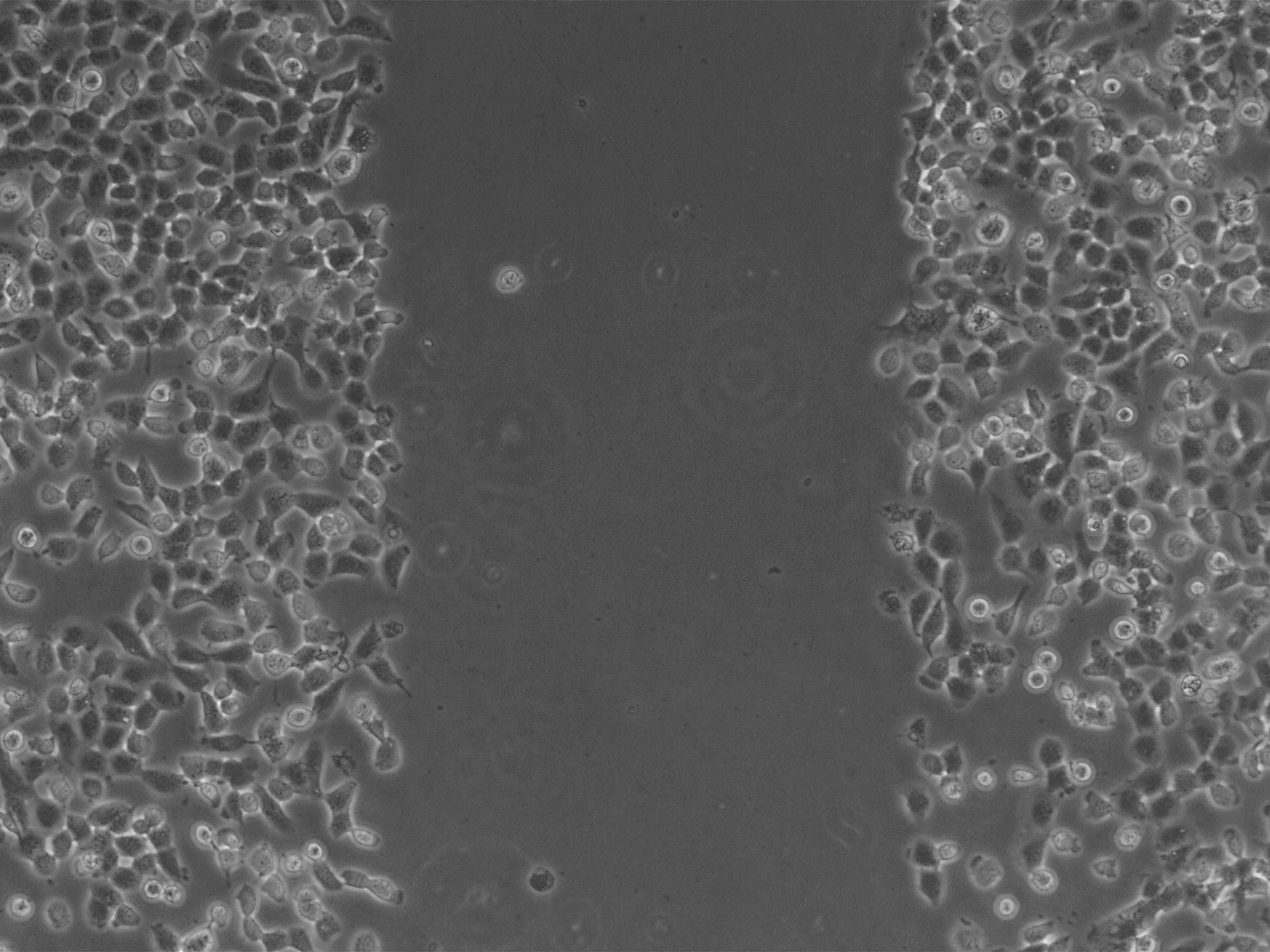

Supplement: Supplemental Information 5 [file peerj-12-16823-s005.zip › H1299/0h/D2-3.bmp]

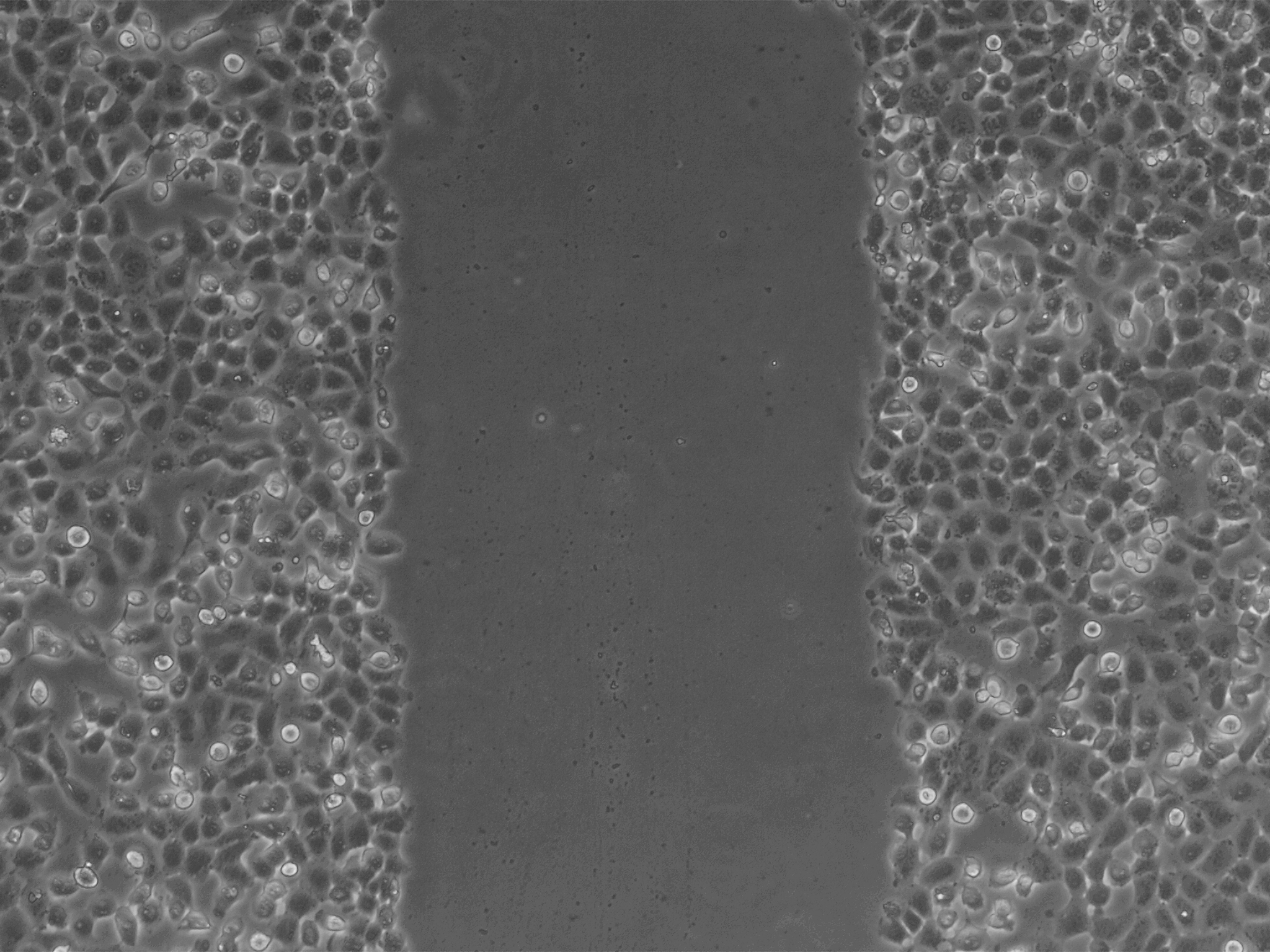

Supplement: Supplemental Information 5 [file peerj-12-16823-s005.zip › H1299/0h/D6-1.bmp]

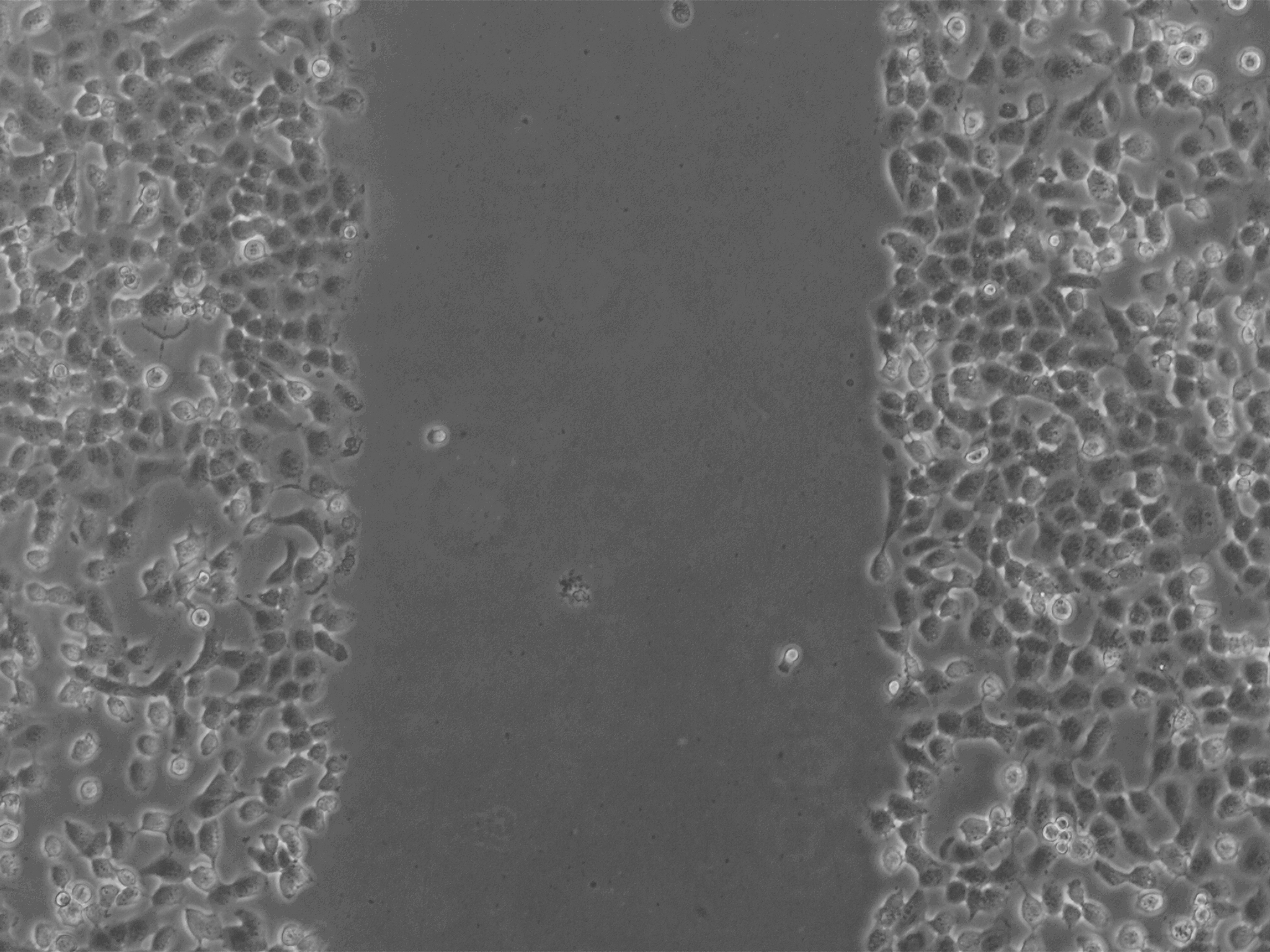

Supplement: Supplemental Information 5 [file peerj-12-16823-s005.zip › H1299/0h/D6-2.bmp]

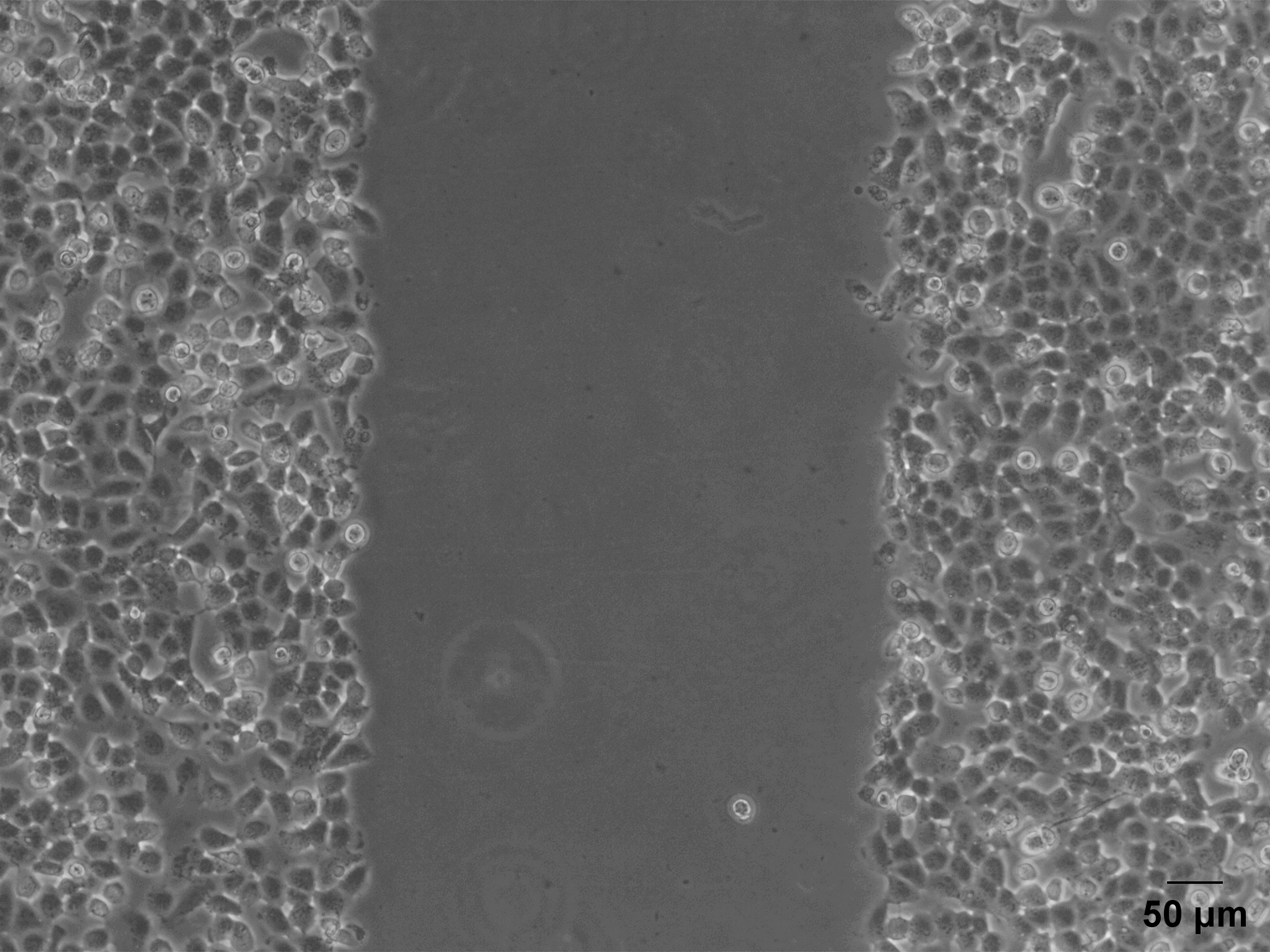

Supplement: Supplemental Information 5 [file peerj-12-16823-s005.zip › H1299/0h/D6-3标尺.tif]

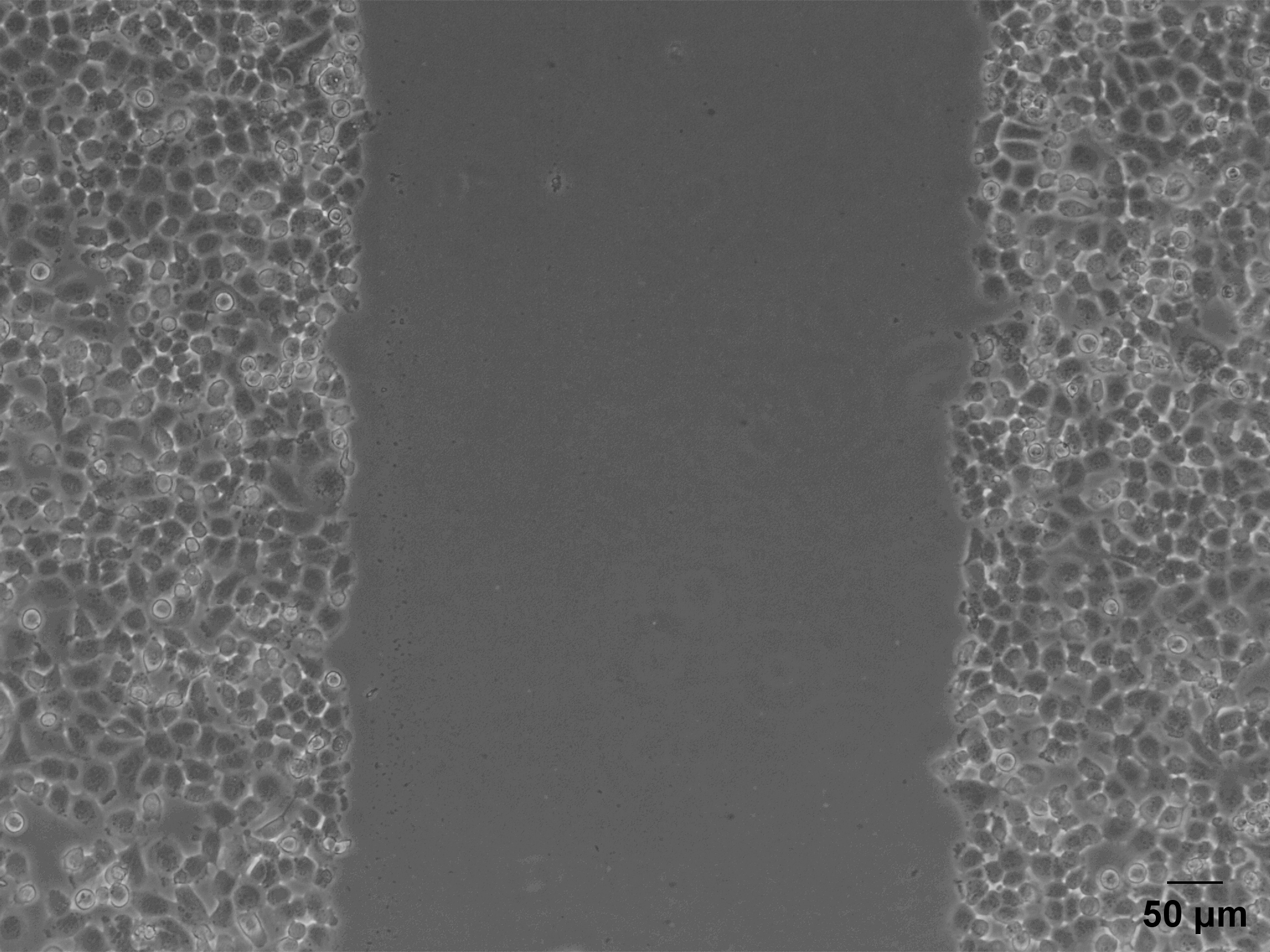

Supplement: Supplemental Information 5 [file peerj-12-16823-s005.zip › H1299/0h/NC-1标尺.tif]

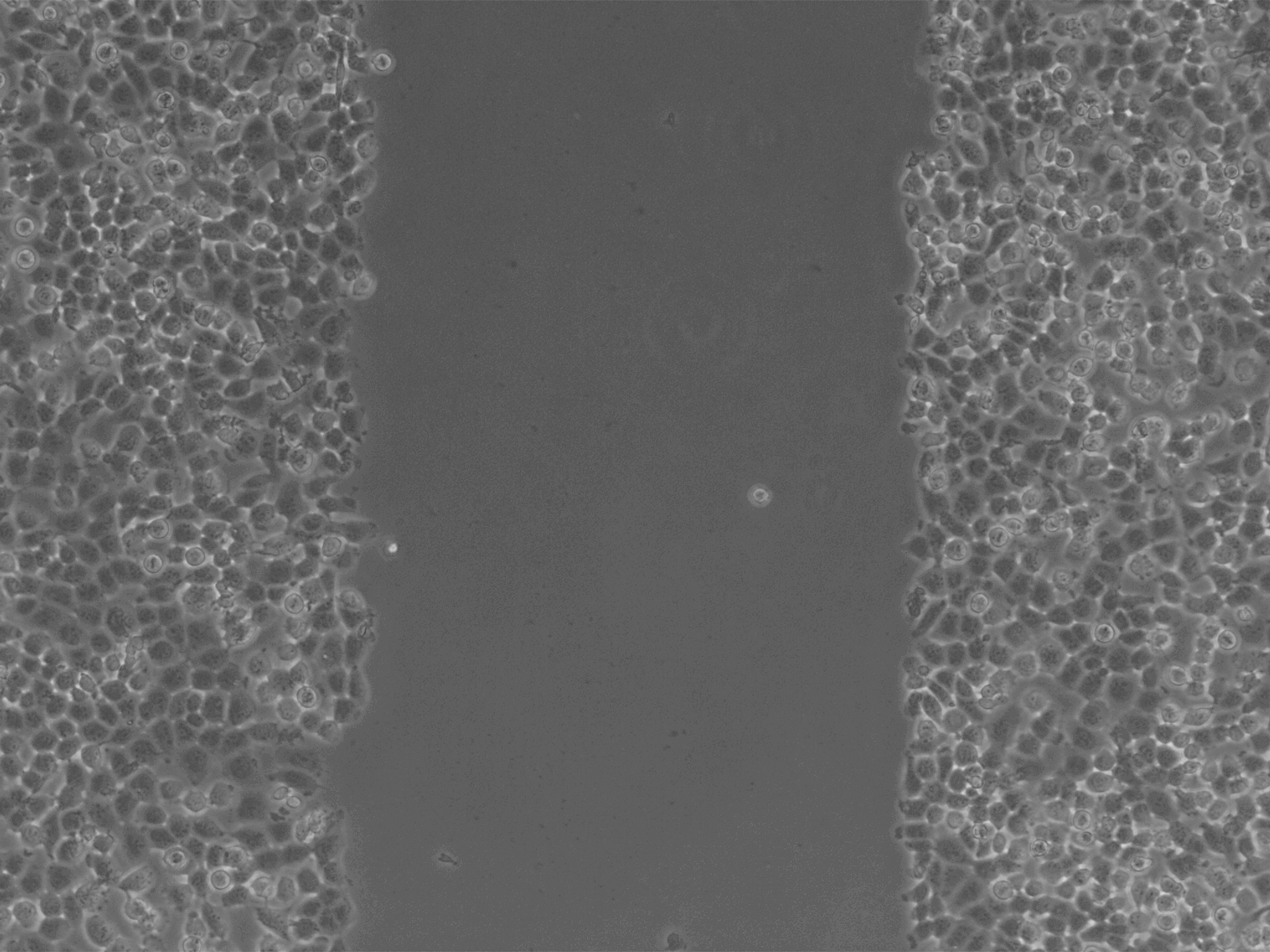

Supplement: Supplemental Information 5 [file peerj-12-16823-s005.zip › H1299/0h/NC-2.bmp]

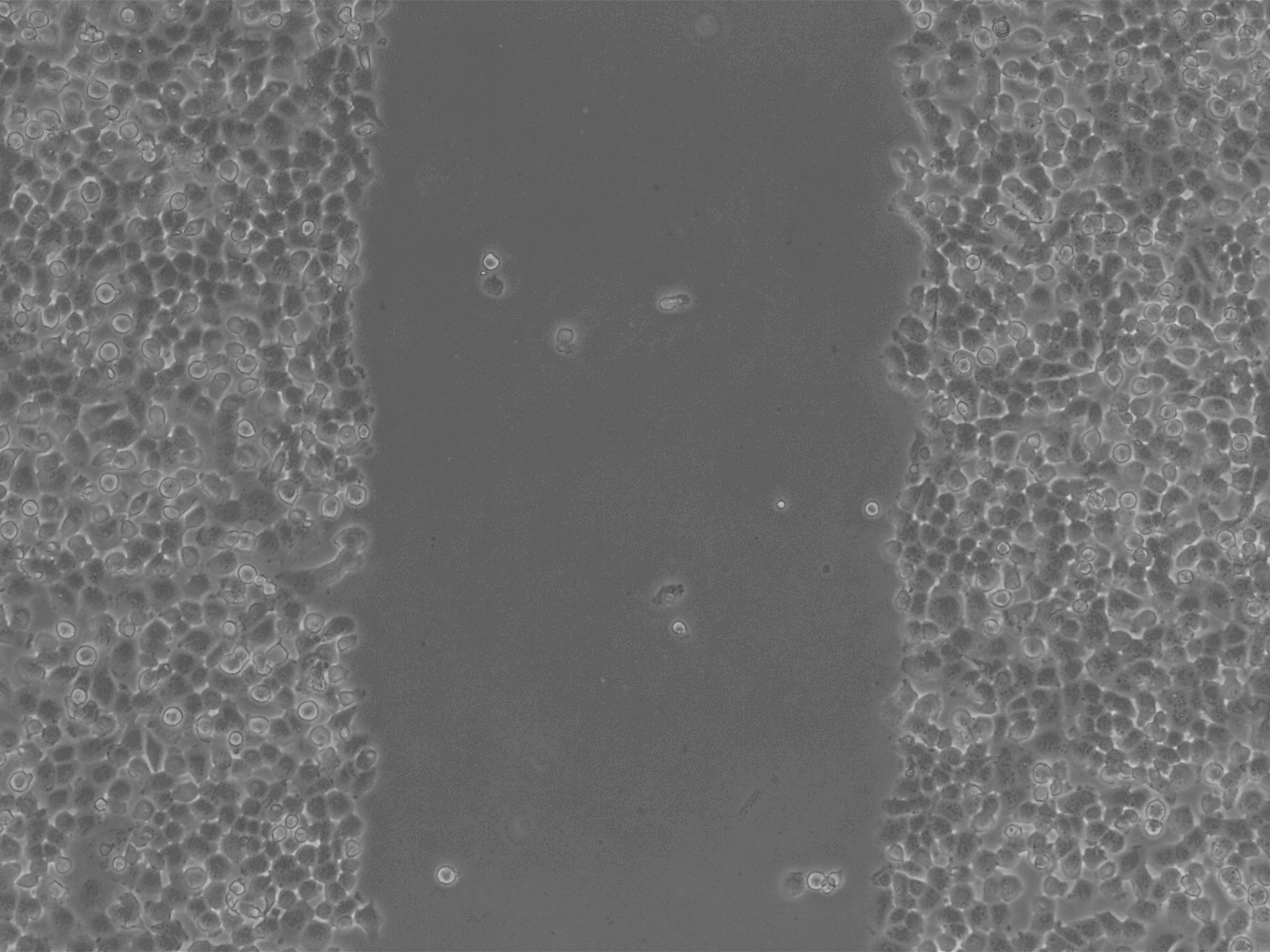

Supplement: Supplemental Information 5 [file peerj-12-16823-s005.zip › H1299/0h/NC-3.bmp]

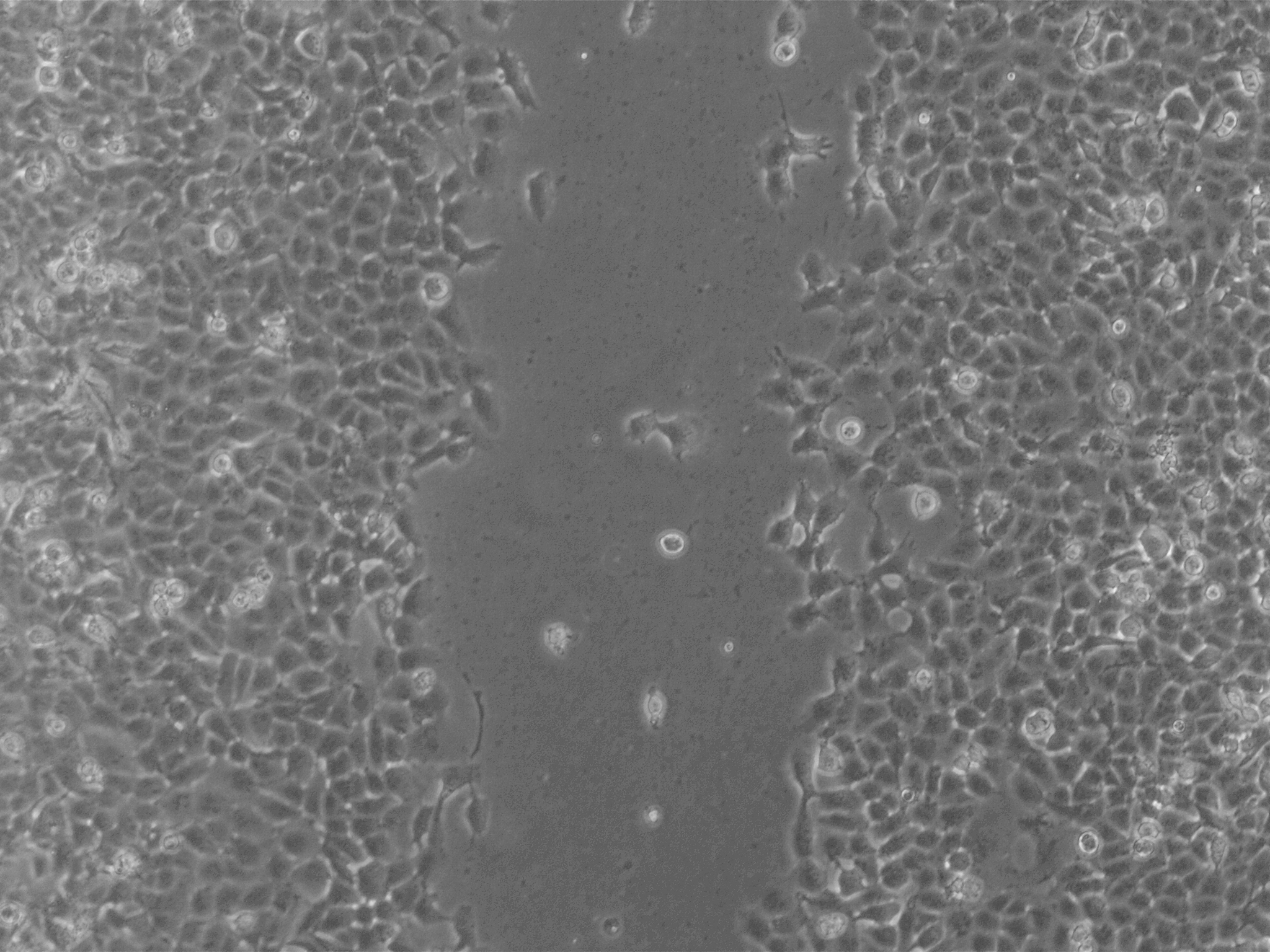

Supplement: Supplemental Information 5 [file peerj-12-16823-s005.zip › H1299/24h/D2-1.bmp]

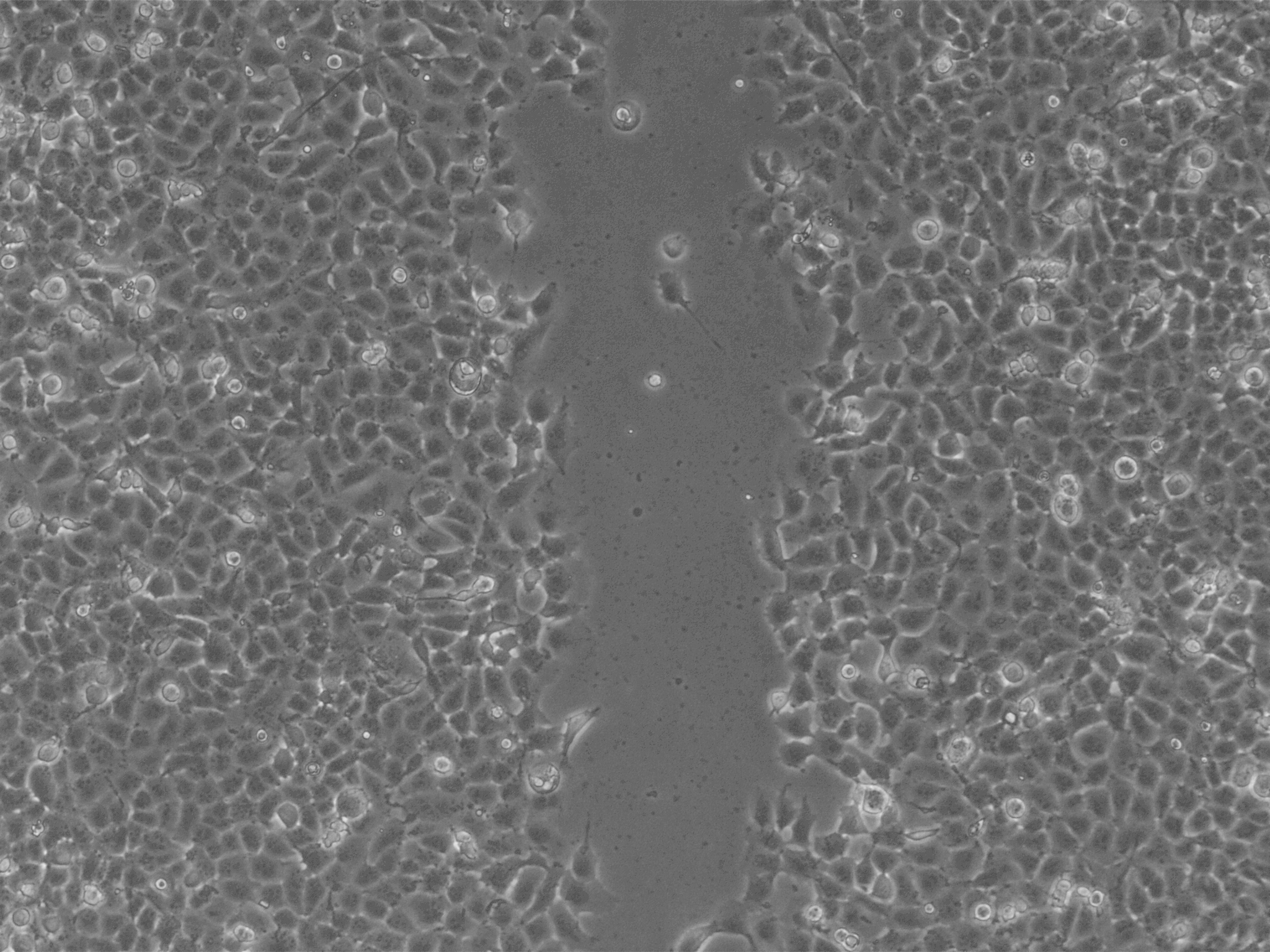

Supplement: Supplemental Information 5 [file peerj-12-16823-s005.zip › H1299/24h/D2-2.bmp]

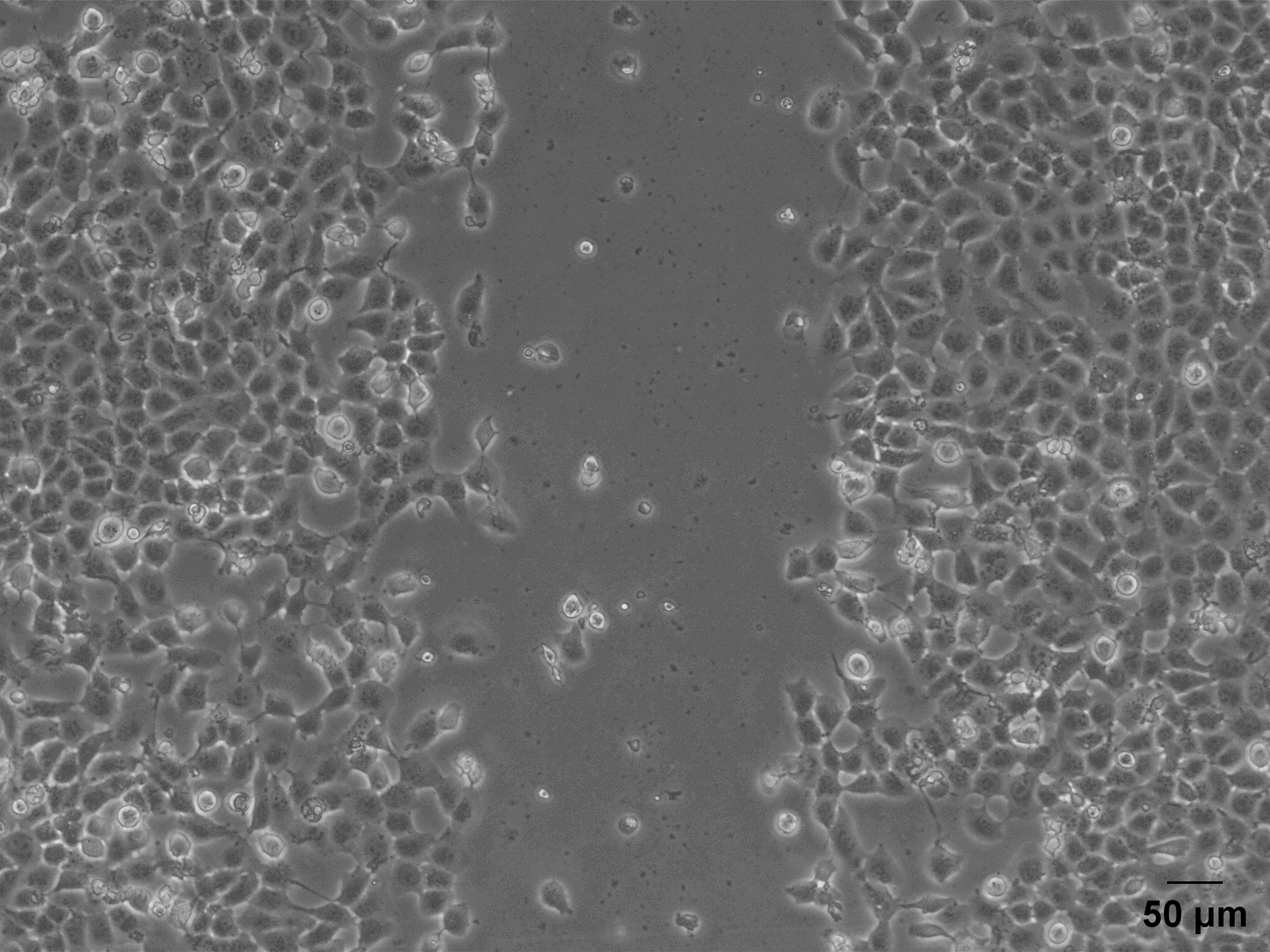

Supplement: Supplemental Information 5 [file peerj-12-16823-s005.zip › H1299/24h/D2-3标尺.tif]

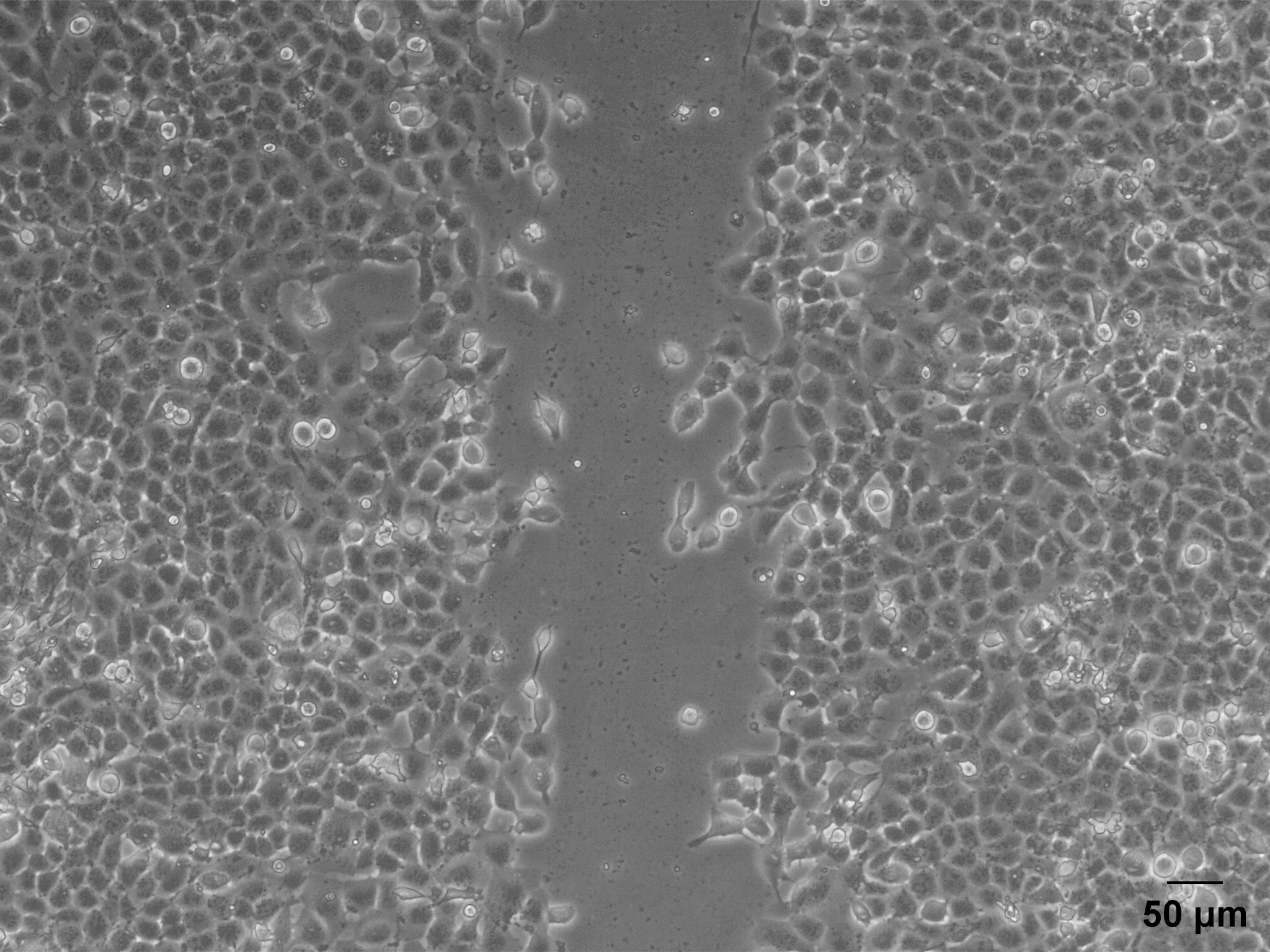

Supplement: Supplemental Information 5 [file peerj-12-16823-s005.zip › H1299/24h/D6-1标尺.tif]

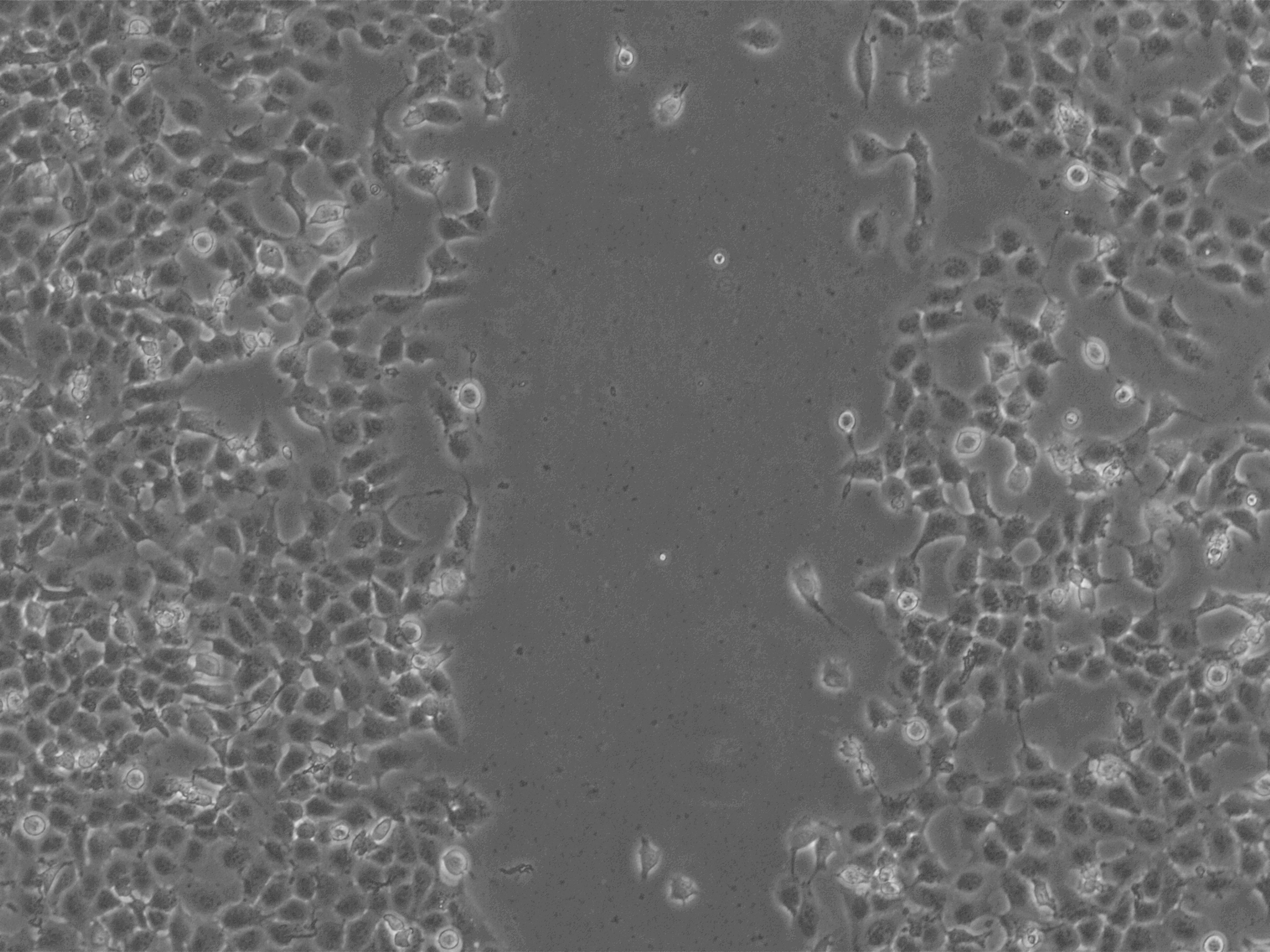

Supplement: Supplemental Information 5 [file peerj-12-16823-s005.zip › H1299/24h/D6-2.bmp]

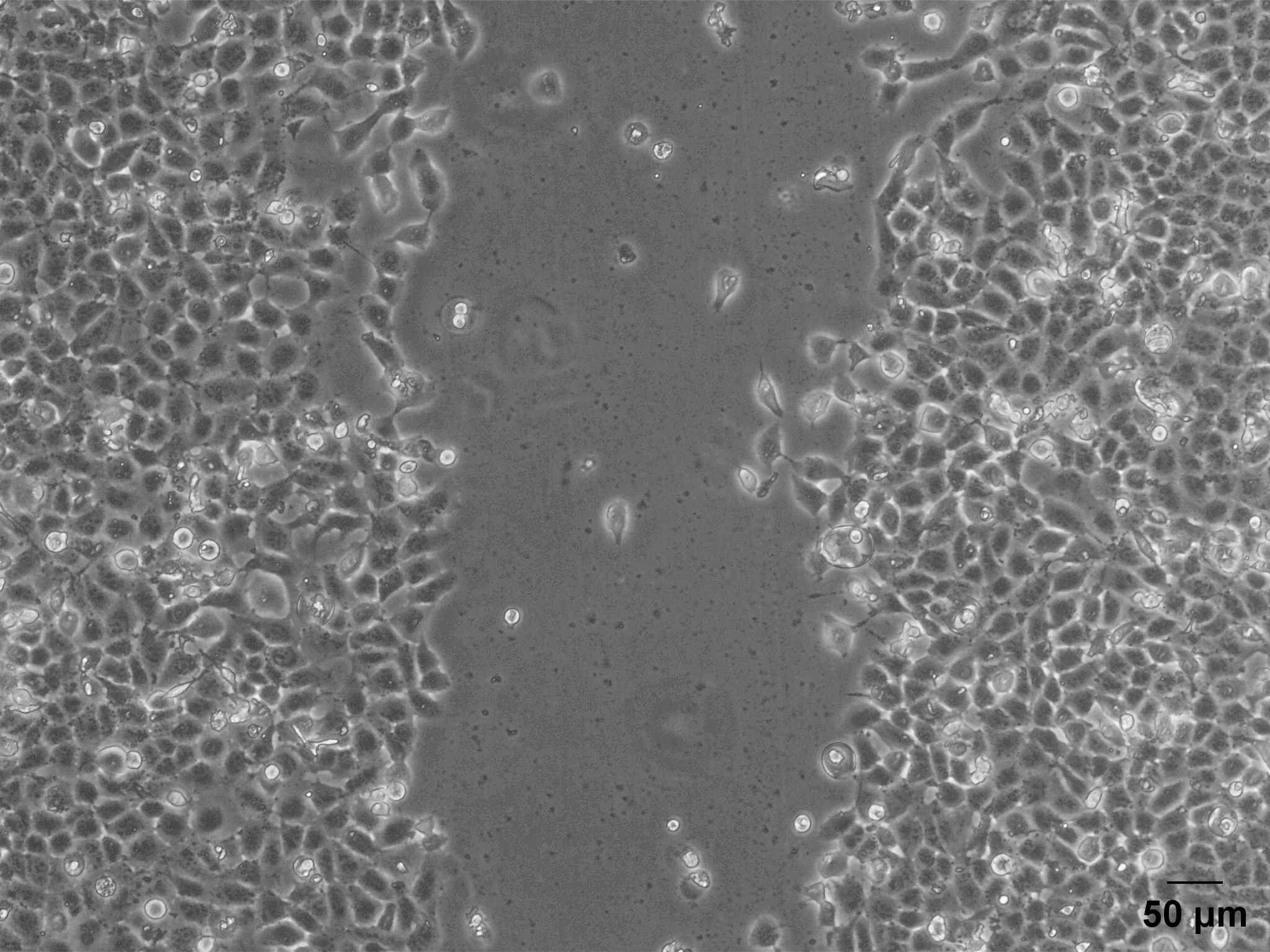

Supplement: Supplemental Information 5 [file peerj-12-16823-s005.zip › H1299/24h/D6-3标尺.tif]

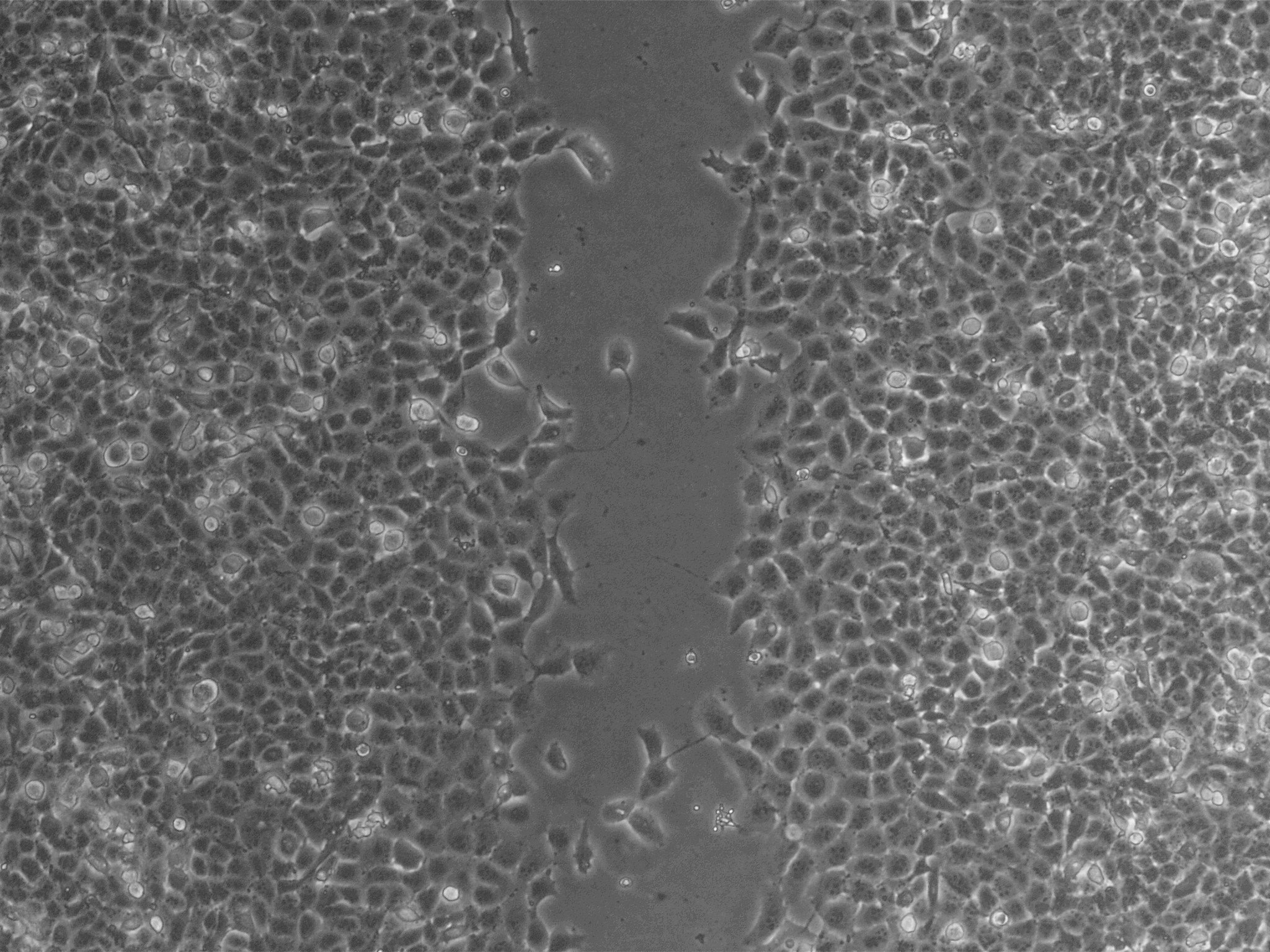

Supplement: Supplemental Information 5 [file peerj-12-16823-s005.zip › H1299/24h/NC-1.jpg]

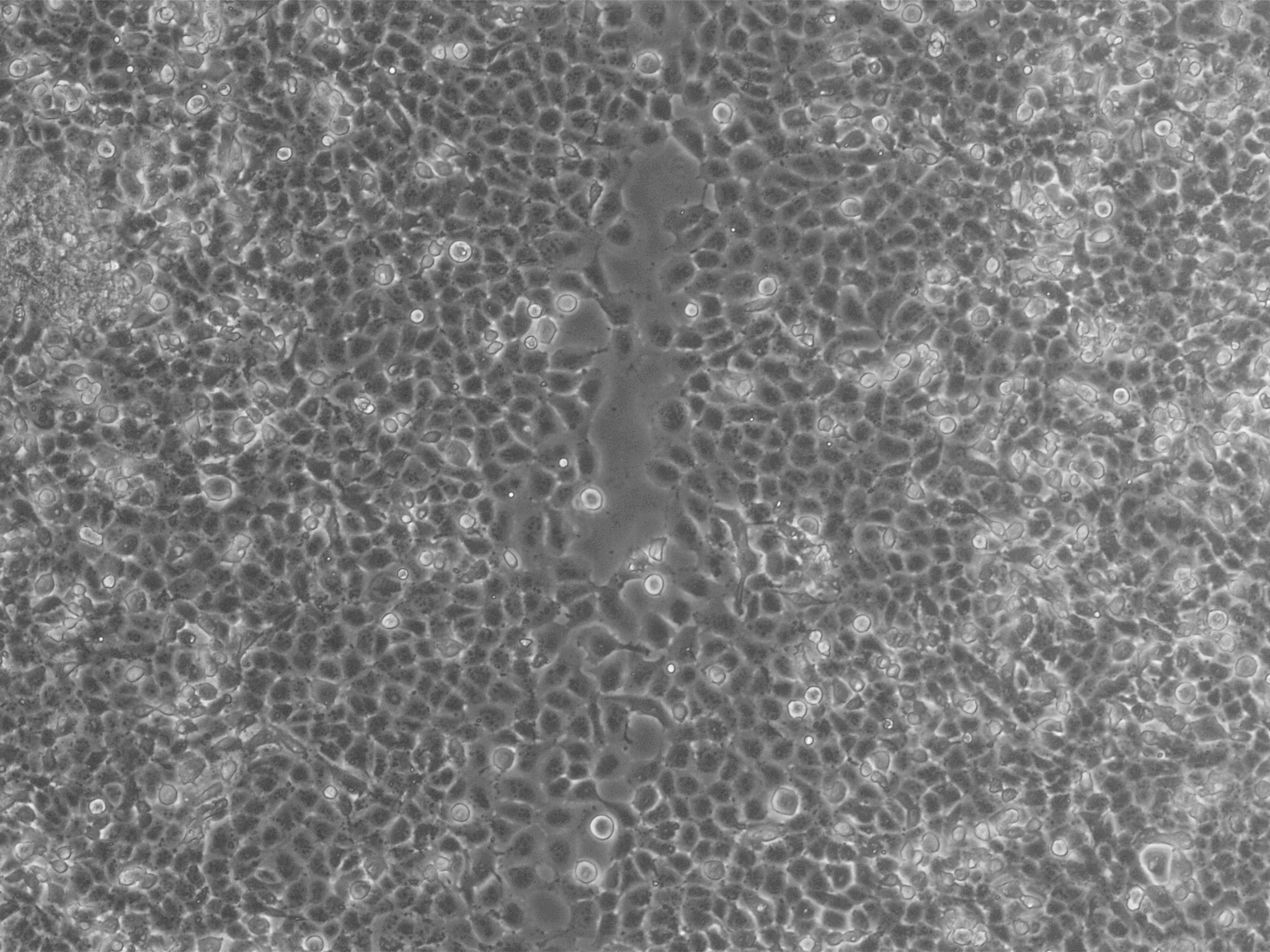

Supplement: Supplemental Information 5 [file peerj-12-16823-s005.zip › H1299/24h/NC-2.jpg]

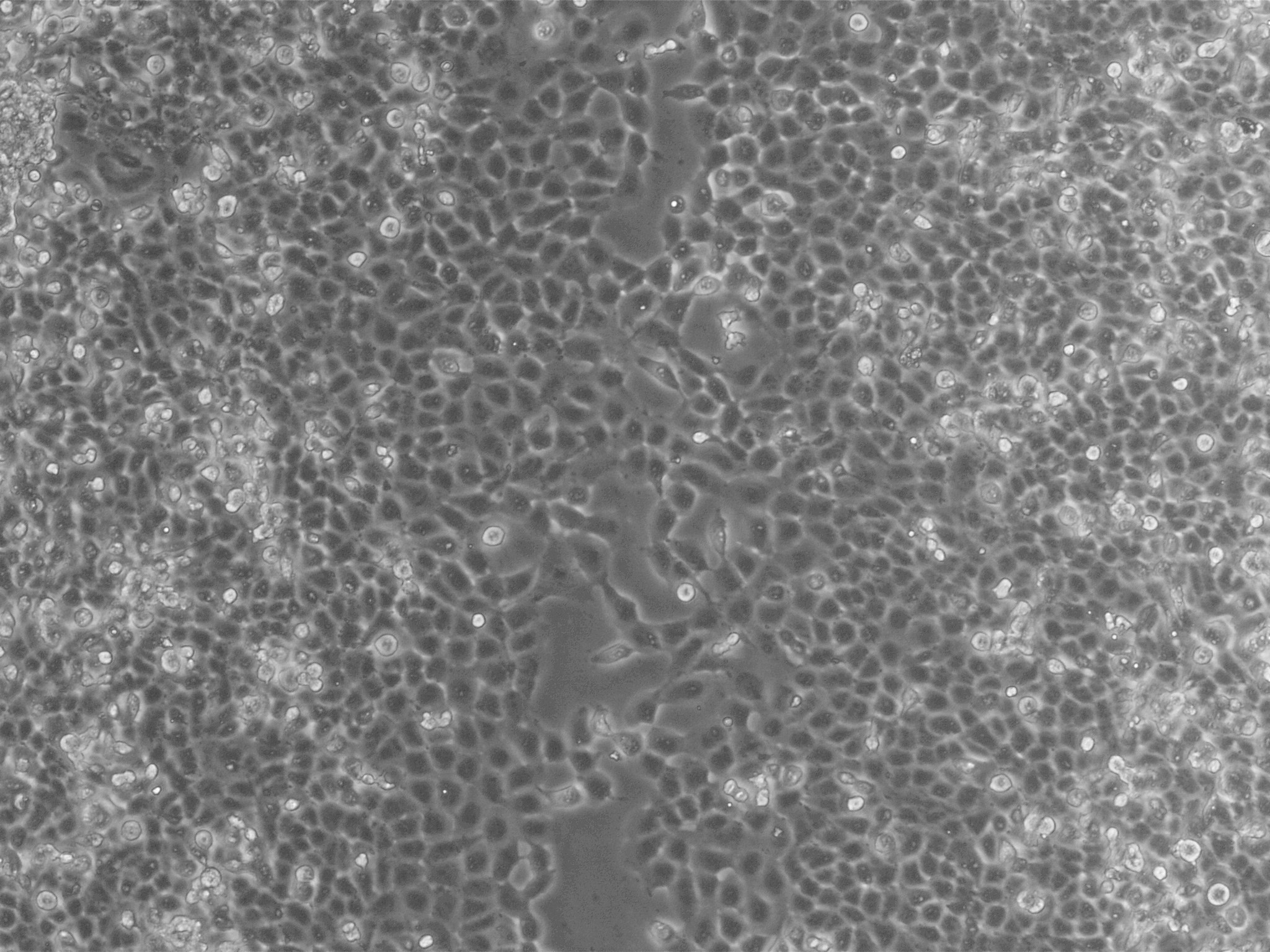

Supplement: Supplemental Information 5 [file peerj-12-16823-s005.zip › H1299/24h/NC-3.jpg]

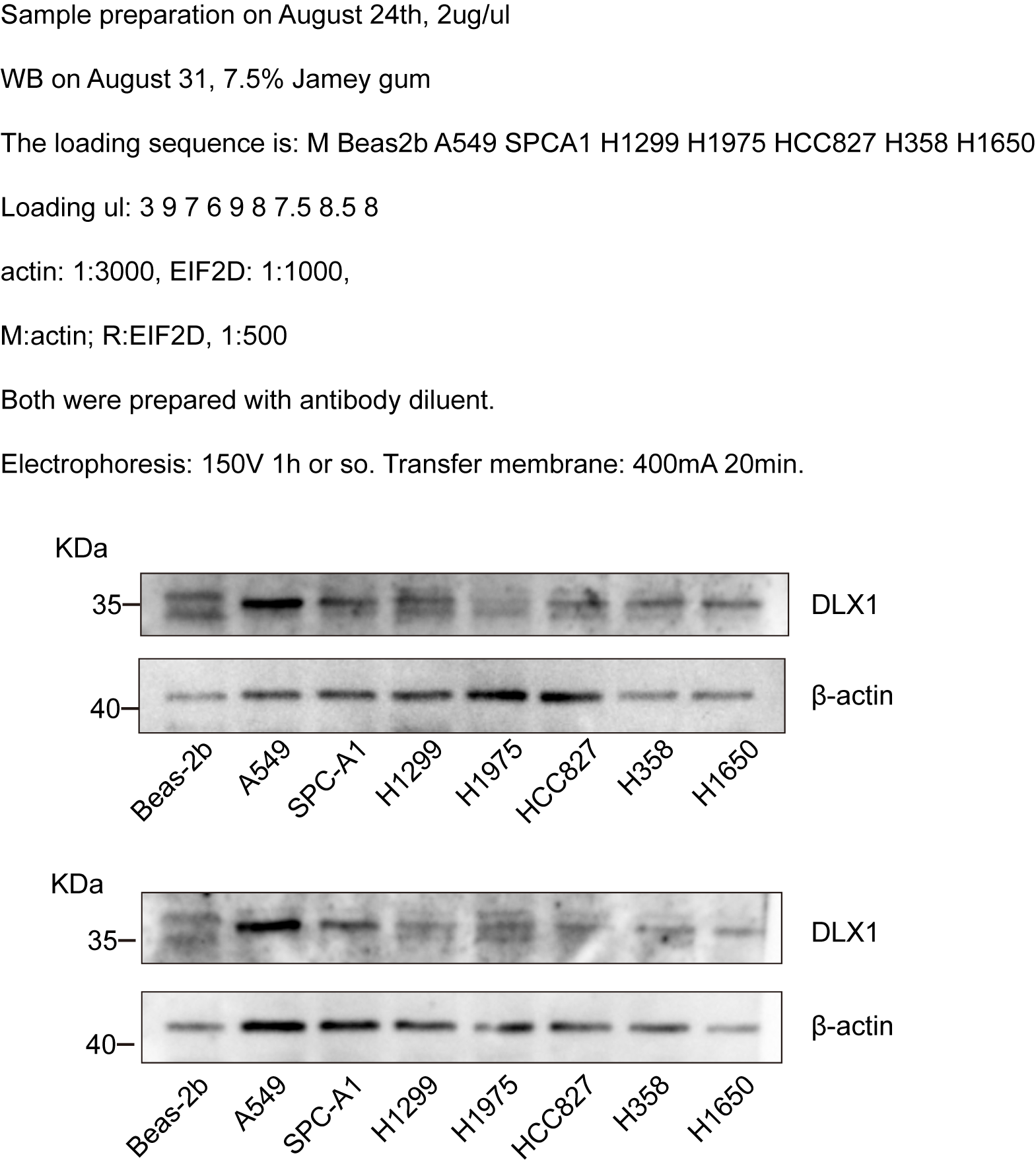

Supplement: Supplemental Information 6 [file peerj-12-16823-s006.png]

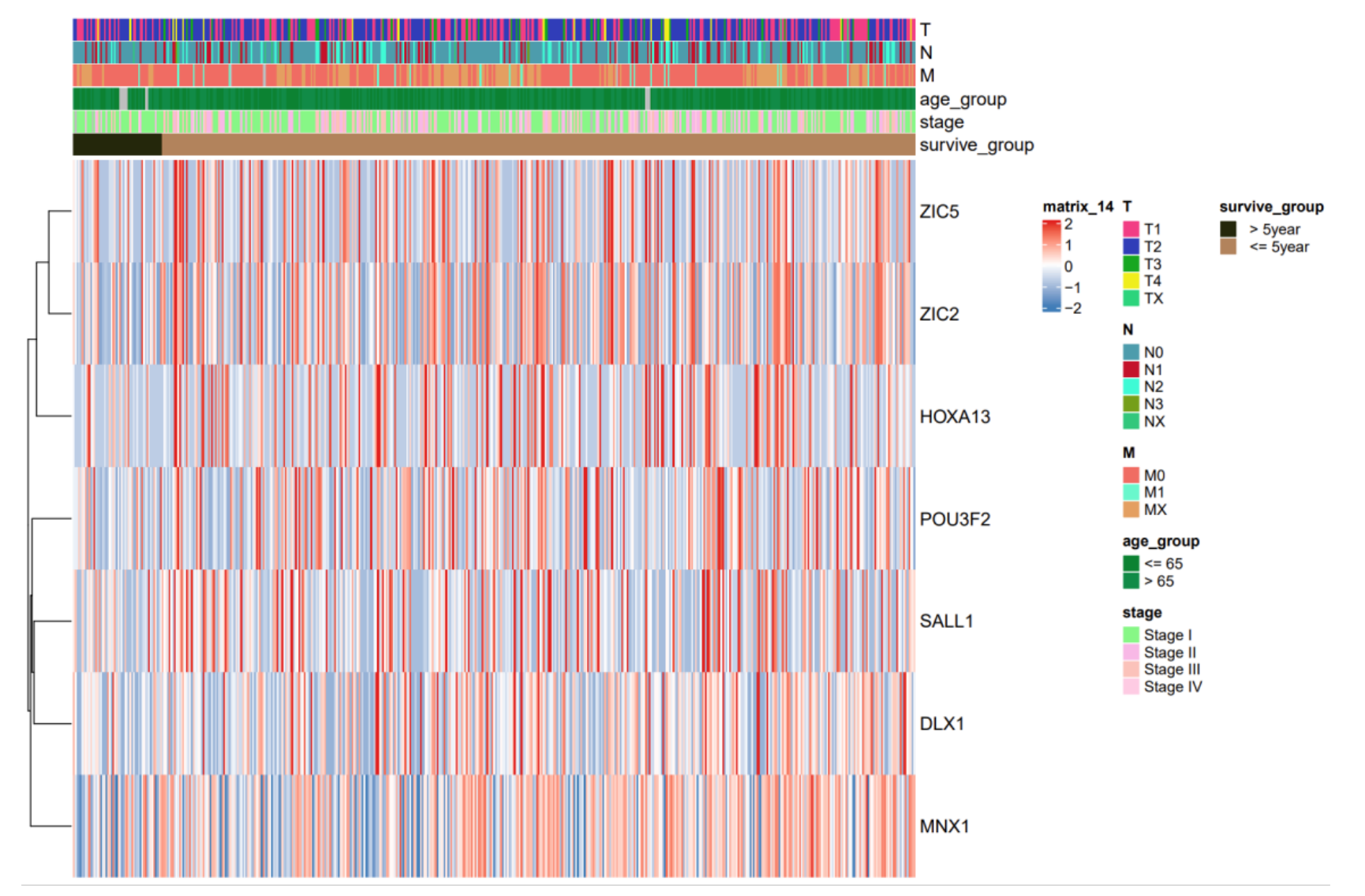

Supplement: Supplemental Information 12 [file peerj-12-16823-s012.png]

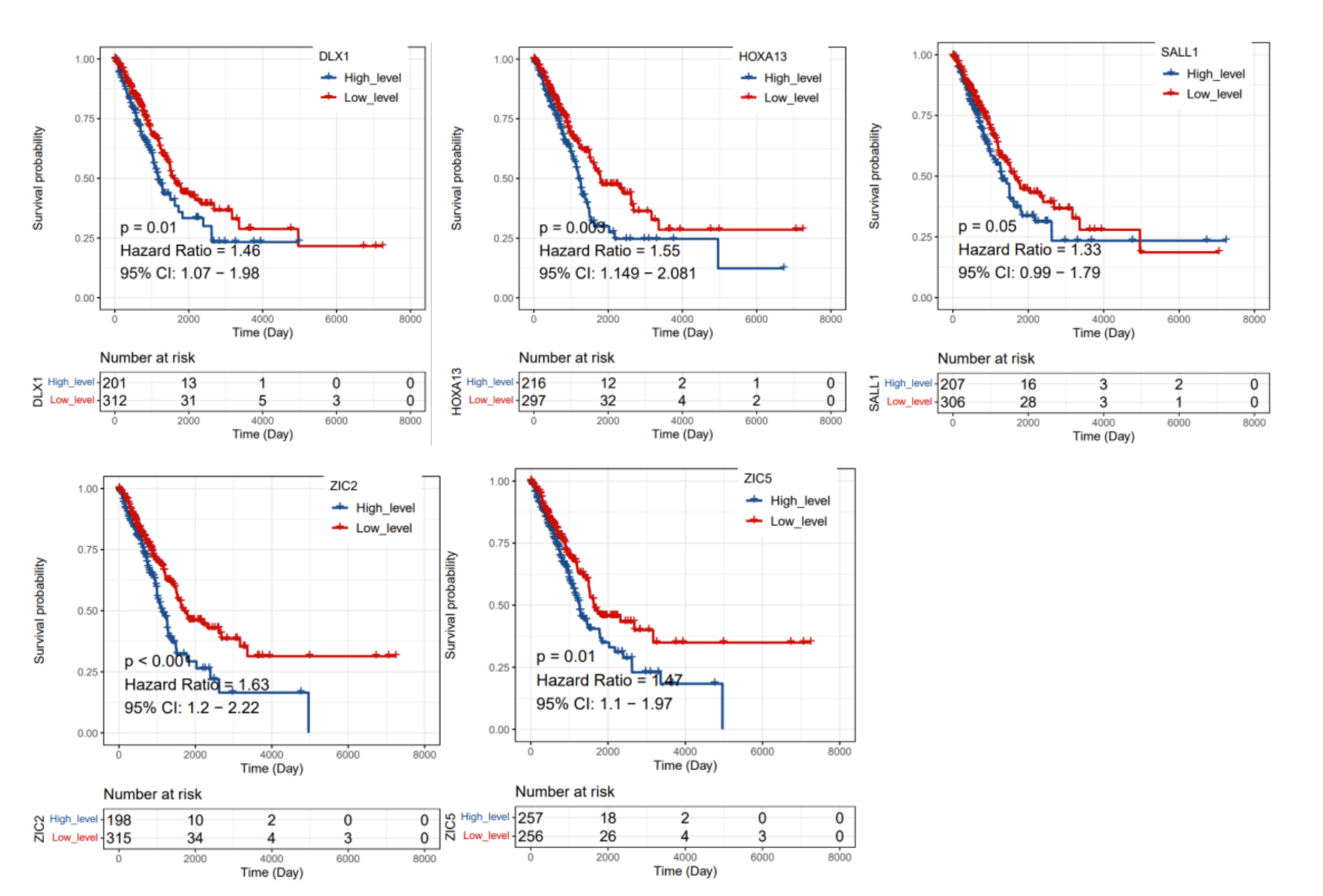

Supplement: Supplemental Information 13 [file peerj-12-16823-s013.png]

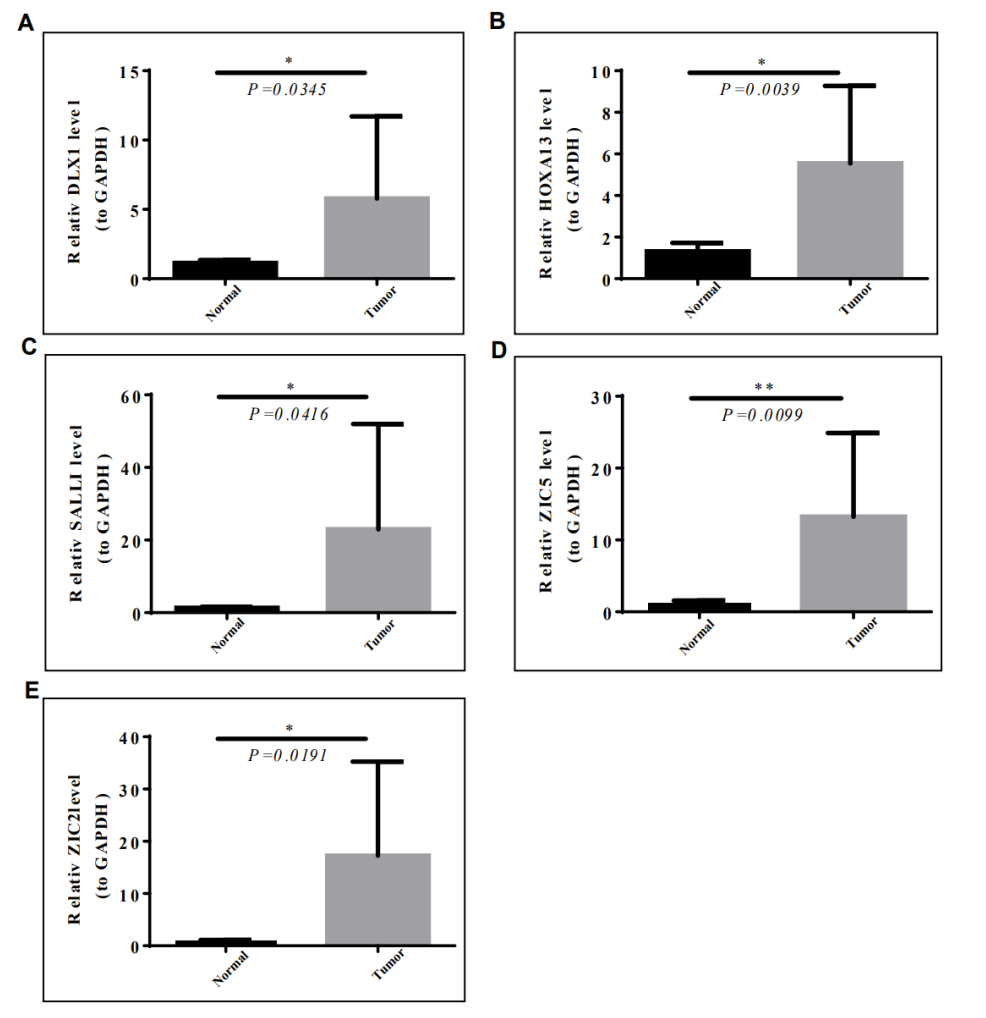

Supplement: Supplemental Information 14 [file peerj-12-16823-s014.png]

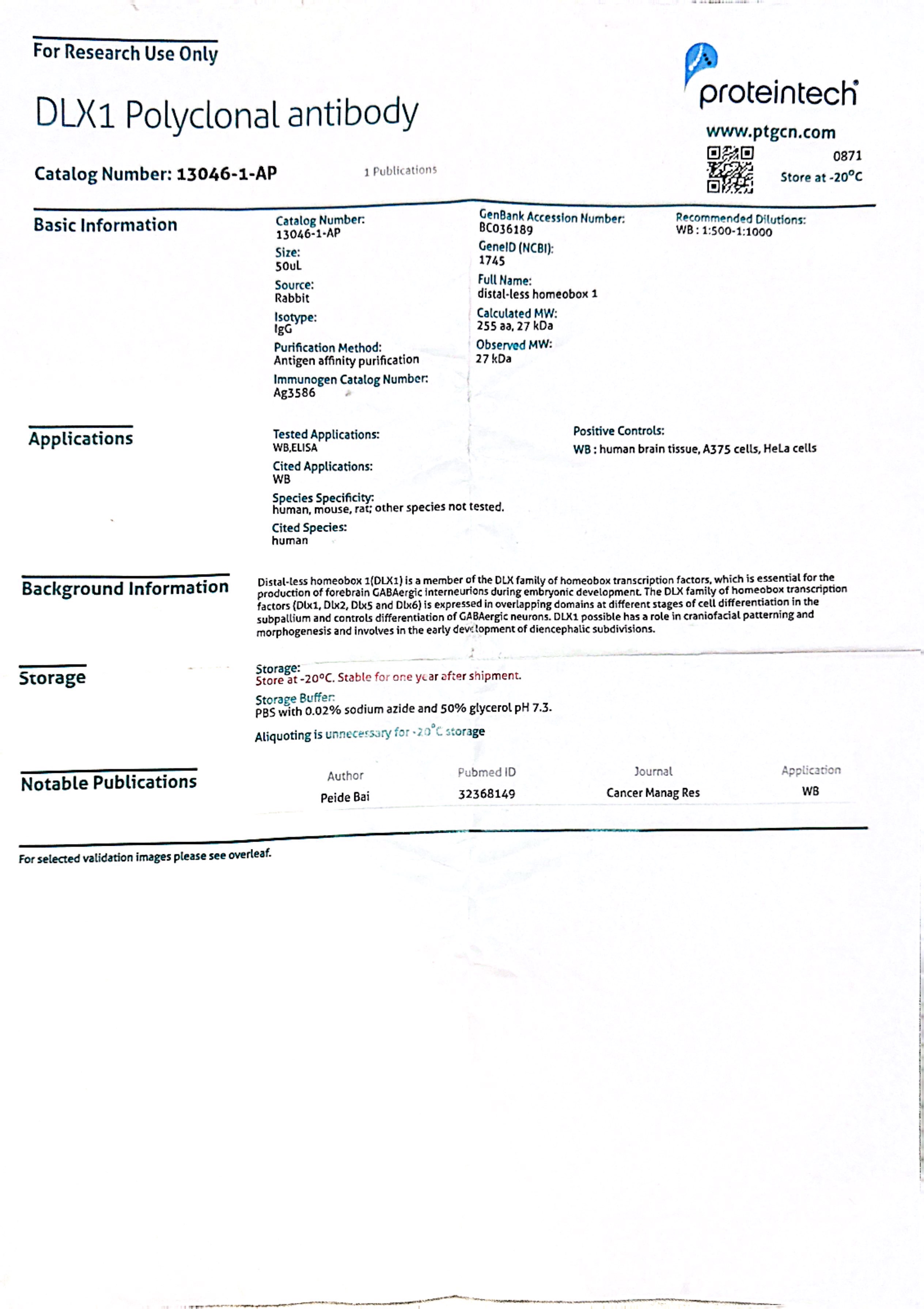

Supplement: Supplemental Information 15 [file peerj-12-16823-s015.jpg]

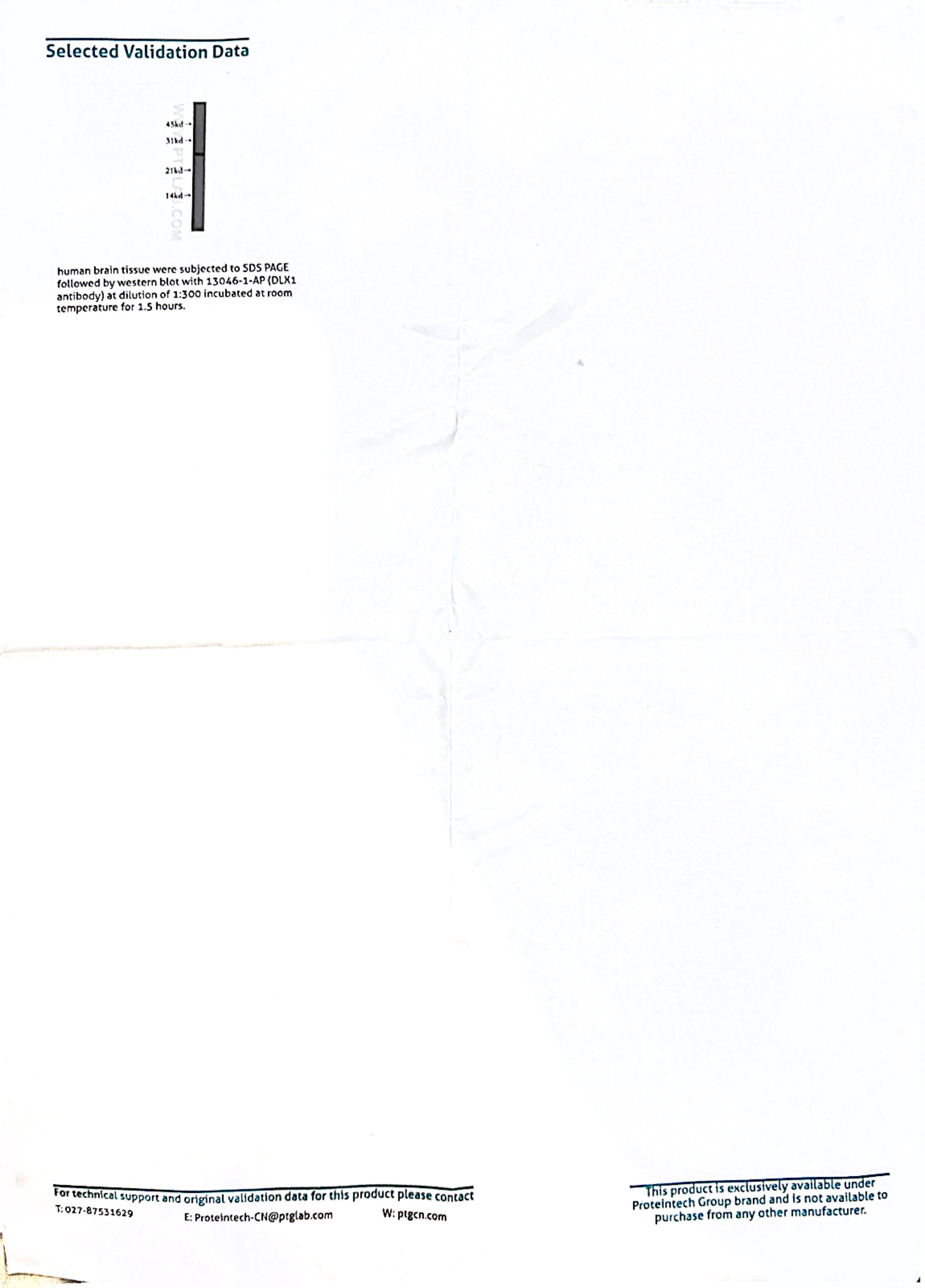

Supplement: Supplemental Information 16 [file peerj-12-16823-s016.jpg]
